# Modeling and Strength Calculations of Parts Made Using 3D Printing Technology and Mounted in a Custom-Made Lower Limb Exoskeleton

**DOI:** 10.3390/ma17143406

**Published:** 2024-07-10

**Authors:** Szczepan Śpiewak, Wiktoria Wojnicz, Jan Awrejcewicz, Magdalena Mazur, Michał Ludwicki, Bartosz Stańczyk, Bartłomiej Zagrodny

**Affiliations:** 1 Institute of Mechanics and Machine Design, Faculty of Mechanical Engineering and Computer Science, Czestochowa University of Technology, ul. Dabrowskiego 73, 42-201 Czestochowa, Poland; 2 Faculty of Mechanical Engineering and Ship Technology, Gdansk University of Techology, ul. Narutowicza 11/12, 80-233 Gdansk, Poland; wiktoria.wojnicz@pg.edu.pl; 3 Department of Automation Biomechanics and Mechatronics, Lodz University of Technology, ul. Stefanowskiego 1/15, 90-924 Lodz, Poland; jan.awrejcewicz@p.lodz.pl (J.A.); michal.ludwicki@p.lodz.pl (M.L.); bartosz.stanczyk@p.lodz.pl (B.S.); bartlomiej.zagrodny@p.lodz.pl (B.Z.); 4 Department of Production Engineering and Safety, Czestochowa University of Technology, ul. Dabrowskiego 69, 42-201 Czestochowa, Poland; magdalena.mazur@pcz.pl

**Keywords:** exoskeleton, finite element method, ABS, orthotropic material, modeling, numerical study

## Abstract

This study is focused on the application of 3D-printed elements and conventional elements to create a prototype of a custom-made exoskeleton for lower limb rehabilitation. The 3D-printed elements were produced by using Fused Deposition Modeling technology and acrylonitrile butadiene styrene (ABS) material. The scope of this work involved the design and construction of an exoskeleton, experimental testing of the ABS material and numerical research by using the finite element method. On the basis of the obtained results, it was possible to deduce whether the load-bearing 3D-printed elements can be used in the proposed mechanical construction. The work contains full data of the material models used in FEM modeling, taking into account the orthotropic properties of the ABS material. Various types of finite elements were used in the presented FE models. The work is a comprehensive combination of material testing issues with the possibility of implementing the obtained results in numerical strength models of machine parts.

## 1. Introduction

Fused Deposition Modeling (FDM) technology is used to produce 3D-printed elements [1]. This approach uses a polymer filament (e.g., acrylonitrile butadiene styrene (ABS)) that is liquefied and, in the form of subsequent layers, are laid using a movable nozzle in accordance with the intended shape of the product. Using this technology, one can produce elements for educational or decoration reasons [2]. Also, this technology can be used to produce elements with complex shapes that can sustain static and fatigue loads [3,4]. However, in this case, the application of 3D-printed elements is limited due to the low mechanical parameters that are key to the 3D printer’s technical possibilities and settings and the mechanical properties of the used filament [5,6,7]. Some reports declare that one can apply a specific method of surface treatment, e.g., laser polishing [8] or heat treatment [9], to obtain elements that can meet the given mechanical requirements.

In the case of the FDM-printed element application in a mechanical system, the mechanical properties should meet the given strength criteria (e.g., yielding strength, tensile strength, compress strength, torsion strength and fatigue strength, etc.) and the stiffness criteria (referring to element dimensions) by considering the given factor of safety. To assess whether a 3D-printed material meets the given requirements, static and fatigue mechanical tests have to be performed using specially prepared samples. There are some reports describing the results of mechanical testing of elements printed using ABS material, namely, the static tensile test [10], impact test [11] and fatigue test [12]. Moreover, typical industrial tests of this material for special load conditions are described in [13]. Research on the mechanical properties of glued joints has recently been carried out [14]. What is more important is a spatial configuration of 3D-printed element layers that are supposed to carry external loadings. 

Certain functional properties of a virtually designed component may be controlled with the use of developed models representing the phenomena occurring in this component. Conducting a simulation of the designed object’s behavior under given conditions gives the designer information about the possibilities of using the tested element in the device as well as determines the direction of the necessary modifications. However, a more complete check of the functional properties of the designed device is possible after creating a prototype of construction.

It is worth noting that there are only a few reports describing the application of a 3D-printed element produced to sustain a specific external load [15,16,17,18]. Moreover, there is a noticeable lack of publications presenting a comprehensive study describing the design, modeling and numerical research conducted to test whether the assumed mechanical criteria are met.

In strength analyses, it is important to take into account the interactions between all parts, especially those made of different materials; therefore, to conduct numerical analysis aiming to assess the strain and stress state developed in the tested element under external load influence, one can use a finite element method (FEM) (Supplementary Section S4). The task of testing the strength of parts made using 3D printing was set for the authors of this work. The analyzed object, in which some parts were made using the mentioned technology, is a prototype of a custom-made exoskeleton for lower limb rehabilitation.

The basic problem of FEM modeling concernsthe determination of the method of discretization of the exoskeleton and defining the boundary conditions of the model that should consider real fixation and loadings (Sections S5–S7), and the numerical task should not be too large. However, the main problem was the choice of the type of analysis due to the effects of kinematic nonlinearity and material nonlinearity of the object. It is not difficult to prove that the solids of the exoskeleton will experience large displacements and large rotations. However, the larger problem was to decide whether to employ small or large deformations of the material. The last issue is determining the parameters of the constitutive law as a component of the stiffness matrix in finite elements, especially since the studies [9,15,19] state that FDM-printed elements have to be implemented by using an orthotropic material model. For this purpose, it is necessary to carry out laboratory tests that will allow one to adjust the appropriate material model for the finite elements representing the printed parts. Reference [10] indicates that this problem can be solved by printing samples with a rectangular cross-section shape in three different configurations (relative to the printer’s working plane) and then performing a static tensile test. The authors decided to produce samples with a circular cross-section and print them in two configurations (horizontal and vertical). These samples were subjected to static tensile and torsion tests. It was assumed that such tests would allow for checking whether the material exhibits orthotropy and, at the same time, whether it would be possible to directly obtain Young’s modulus, the shear modulus and Poisson’s ratio. Taking into account the variability of a load carried by an exoskeleton, it was decided to additionally perform fatigue tests on samples using a one-sided repeated cycle.

The analyzed exoskeleton was developed based on a study [20] listing ten different designs of exoskeletons used for the rehabilitation or enhancement of the motor efficiency of the human knee joint, along with descriptions of the type of drive and control unit. A specific type of drive with the use of pneumatic actuators was presented in [21], whereas a propulsion system incorporating electric motors, worm gears and cylindrical gears can be found in Reference [22]. An example of a typical exoskeleton that provides movement stability by means of an integrated propulsion system has been presented [23]. This publication also contains a mathematical description of the kinematics of the object’s motion, taking into account the criteria of stability. A report [24] presents a simplified dimensional optimization of exoskeleton elements involving muscles in the knee joint. The optimization criteria included the reduction in the part mass. Another study [25], however, used a FEM analysis of the internal loading of selected exoskeleton components in the static load range. The FE model of the exoskeleton with a structure similar to the skeletal system of human lower limbs can be found in the publication [26]. These FE models of the exoskeletons can be regarded as highly simplified because the interconnection between the modeled components (contact conditions) was not considered. When selecting the parameters of the exoskeleton drive system, measurement data describing the range of motion of the limbs of a patient with cerebral palsy can be used [27]. However, when designing a system that forces patient movement, the mechanism presented in [28] can be followed.

The ranges of forces generated by human muscles during gait are reported in [22,24]. The motivation of this study was to build a prototype of a lower limb exoskeleton by using (1) 3D-printed elements produced from acrylonitrile butadiene styrene (ABS) and (2) conventional manufacturing elements. The aim of this work was to test whether load-bearing 3D-printed elements met given mechanical criteria. The scope of this study involved designing and building a custom-made prototype of a lower limb exoskeleton and numerical research by using FEM. However, in order for this work to be carried out, it was necessary to simultaneously carry out mechanical tests to determine the parameters of the material model (implemented in the FEM model) for parts made by the 3D printing technique and determine the limits of strength parameters. 

## 2. Materials and Methods

### 2.1. Design

**Figure 1:**
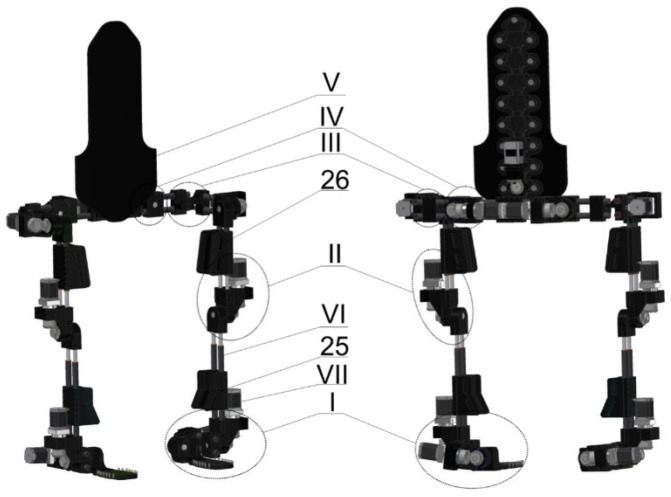
Model of lower limb exoskeleton (anterior view (**left**) and posterior view (**right**)): ankle joint assembly (I), knee joint assembly (II),hip joint assembly (III), pelvis module assembly (IV), spine support (V), spring connectors (VI), driving unit (VII), shank mount (25) and thigh mount (26).

**Figure 2:**
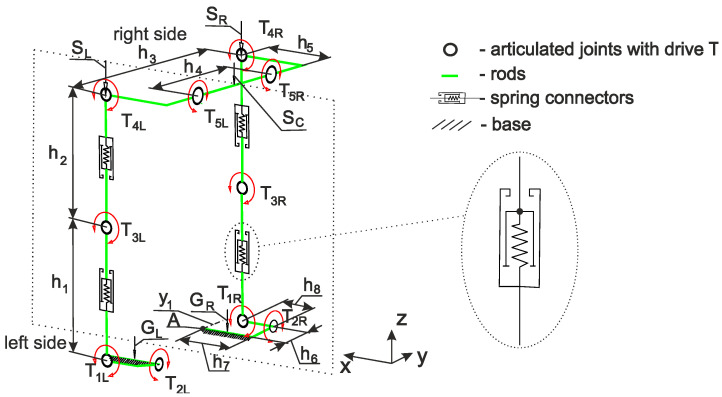
Structural scheme of lower limb exoskeleton prototype (principal dimensions of the model: shin segment *h*_1_ = 485 mm, thigh segment *h*_2_ = 400 mm, frontal right-left hip joint segment *h*_3_ = 760 mm, pubic segment *h*_4_ = 410 mm, lateral hip joint segment *h*_5_ = 265 mm, frontal foot segment *h*_6_ = 120 mm, central foot segment *h*_7_ = 215 mm and lateral foot segment *h*_8_ = 135 mm) (descriptions of other symbols are given in Supplementary Section S1).

A structural scheme describing the function of a custom-made prototype of the lower limb exoskeleton (Figure 1) is given in Figure 2. The model of this prototype was prepared in Autodesk Inventor Professional 2018 software. The exoskeleton dimensions were selected by considering the anthropology of the standard male European (188 cm height) [29,30,31,32]. Selected dimensions (*h*_1_, *h*_2_, *h*_3_, *h*_5_ and *h*_8_) can be adapted in the 10% range. The angular displacements of exoskeleton segments are articulated by torques (generated by electric motors): the right articulatio talocruralis *T*_1*R*_, the right articulatio subtalaris *T*_2*R*_, the left articulatio talocruralis *T*_1*L*_, the left articulatio subtalaris *T*_2*R*_, the flexion/extension of the right knee joint *T*_3*R*_, the flexion/extension of the left knee joint *T*_3*L*_, the flexion/extension of the right hip joint *T*_4*R*_, the flexion/extension of the left hip joint *T*_4*L*_, the abduction/adduction of the right hip joint *T*_5*R*_ and the abduction/adduction of the left hip joint *T*_5*L*_.

**Figure 3:**
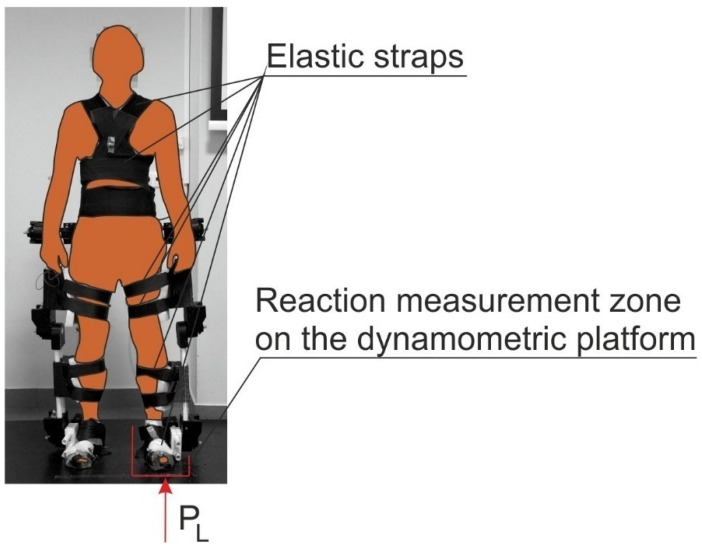
Exoskeleton application.

Due to the fact that the centers of the human joints do not coincide with the centers of exoskeleton joints, spring elements were introduced in both thigh segments and shin segments (Section S2). In practice, a subject is supposed to be fixed to the exoskeleton by using elastic bands (Figure 3).

**Figure 4:**
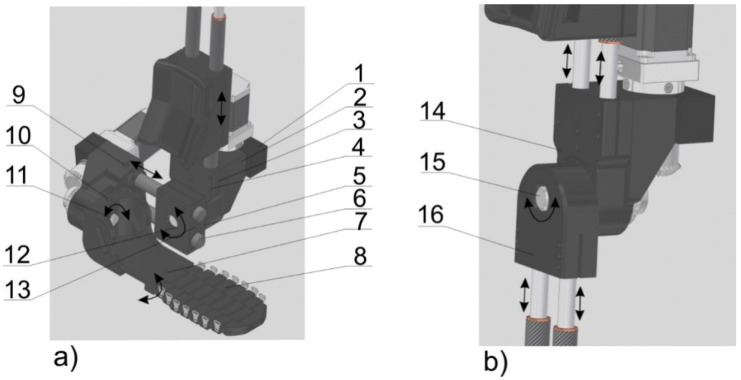
Ankle joint assembly (**a**) and knee joint assembly (**b**). Part names: engine clamp (1), engine mount (2), outer sleeve of the elastic connector (3), screws (4), regulation clamp (5), ankle joint segment connectors (6), heel segment (7), metatarsus segment (8), pins (9, 10 and 13), shafts (11, 12 and 15), upper housing of the inner sleeve of the elastic connector (14) and lower housing of the internal sleeve of the elastic connector (16).

**Figure 5:**
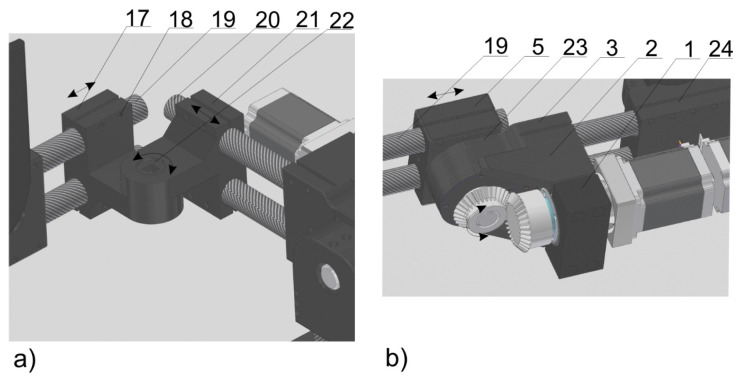
Hip joint assembly (**a**) and pelvis assembly (**b**). Part names: engine clamp (1), engine mount (2), outer sleeve of the elastic connector (3), regulation clamp (5), lateral pelvis regulation clamp (17), screws (18), pelvis segment connectors (19), hip connectors (20), rear pelvis regulation clamp (21), mushroom head bolt (22), cover (23) and main mount (24).

According to the goal of this study, this prototype was built by using elements obtained from conventional manufacturing and 3D printing elements 1, 2, 3, 5, 7, 8, 14, 16, 17, 21, 23, 24, 25 and 26 (Figure 1, Figure 4 and Figure 5).

**Figure 6:**
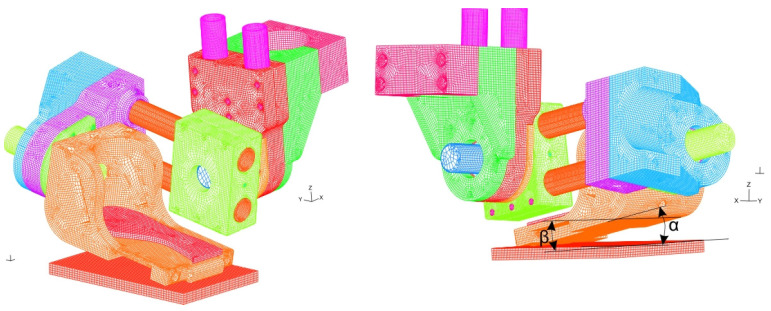
Mesh model of ankle joint assembly: *α* [deg]—angle between the heel segment and the ground measured in the *yz* sagittal plane; *β* [deg]—angle between the heel segment and the ground measured in the *xz* frontal plane.

The motor of the ankle joint is between the engine clamp (1) and the engine mount (2) (Figure 4a). The set of elastic connectors is clamped through the casing of the outer sleeve of the elastic connector (3). Elements 1, 2 and 3 are connected to each other by screws (4). Flexion/extension in the ankle joint is accomplished by rotation of a shaft (12) connected via the pin (13) to the regulation clamp (5). This regulation clamp (5) allows the size of the whole ankle joint segment to be adjusted. An eversion/inversion movement of the foot is realized by the rotation of a shaft (11) coupled by a pin (10) to the heel segment (7). The metatarsus segment (8) is responsible for the free movement of the toes. The size of the metatarsus segment (8) is adjusted to the size of the patient’s foot by adding/removing individual links. A mesh model of the ankle joint assembly is shown in Figure 6.

**Figure 7:**
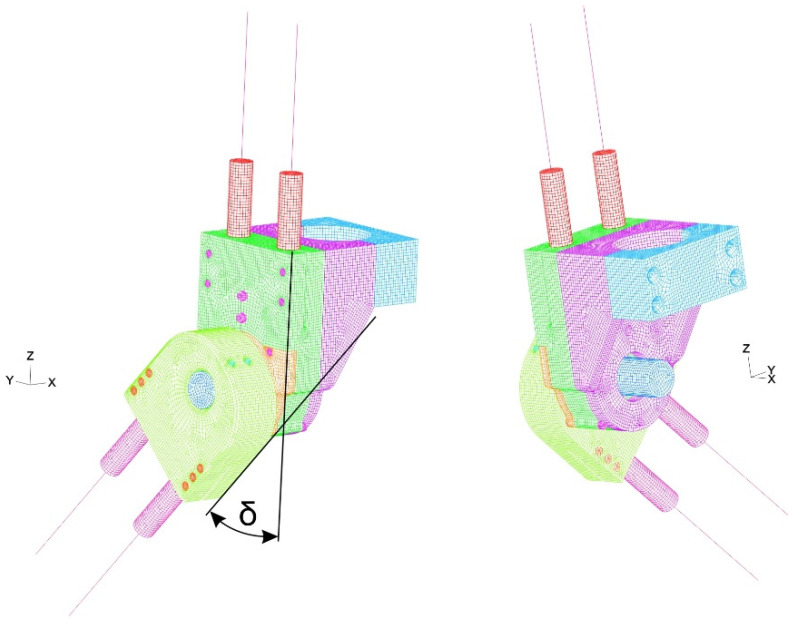
Mesh model of knee joint assembly.

The movement in the knee joint is realized by the assembly shown in Figure 4b. This structure includes the upper housing of the inner sleeve of the elastic connector (14) and the lower housing of the internal sleeve of the elastic connector (16). These elements are mounted on the shaft (15). The rotational flexion/extension movement is realized by a pivot connection (15). A mesh model of the knee joint assembly is shown in Figure 7.

**Figure 8:**
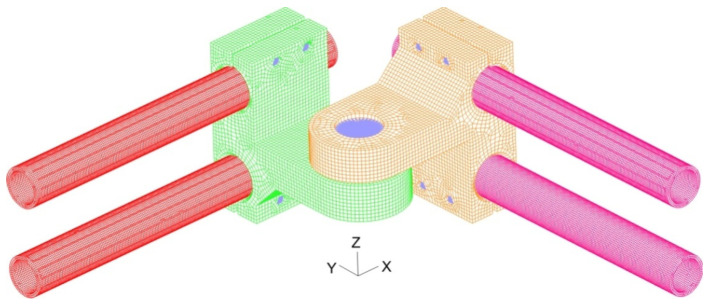
Mesh model of hip joint assembly.

The hip joint assembly is shown in Figure 5a. This system has a regulation clamp (17) mounted on pelvis segment connectors (19), a regulation clamp (21) and hip connectors (20). The connection between parts 17–21 is realized by screws (18). A mushroom head bolt (22) connects two parts, 17 and 21. A mesh model of the hip joint assembly is shown in Figure 8.

The pelvis assembly is shown in Figure 5b. This structure includes the cover (23) that secures the bearing between the shaft and a part (3). The pelvis assembly is connected to the hip joint assembly by the main mount (24). The spine support assembly (V) (Figure 1) supports the upper part of the body by using elastic straps (Figure 3). 

A detailed description of the driving unit is given in Section S3.

FEM analysis of the ankle joint assembly, knee joint assembly and hip joint assembly was conducted in ADINA [30] software. Interactions between elements of each assembly were determined by defining boundary conditions influenced by the lower limb of the patient during balancing. A detailed description of the FEM models is given in Sections S5–S7.

### 2.2. Materials

The data presented in [9,19] indicate that ABS material can be described by the orthotropy material model, and it is influenced by the direction in which subsequent layers are printedin the printing process. That is why values of internal forces in the layer fibers depend on their orientation. Based on References [1,2,3,4,5,6,7,8,9,19], it can be concluded that a large number of parameters that can be set in 3D printers cause the mechanical properties of the printed parts to depend on these settings. Therefore, in order to identify the material model necessary in numerical simulations of loads on exoskeleton parts as precisely as possible, it was decided to carry out mechanical tests on ABS material samples produced in the conditions of the production of the exoskeleton parts.

**Figure 9:**
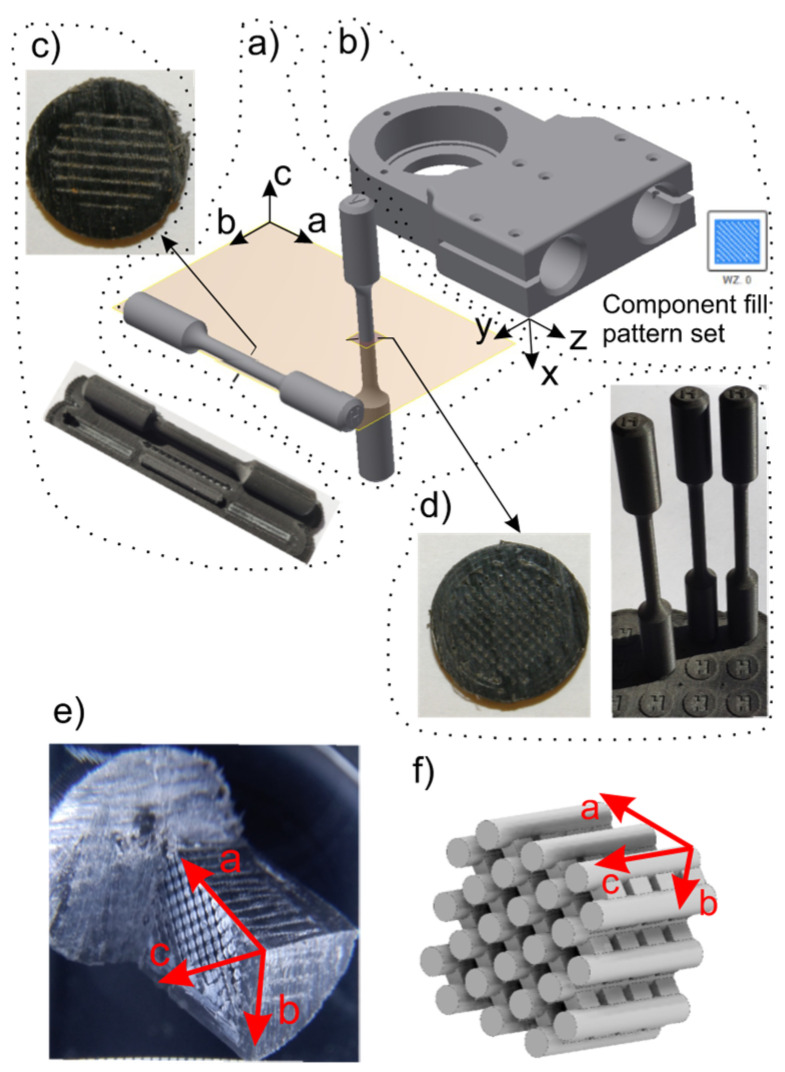
Orientation of the main plane of the 3D-printed samples; chosen 3D-printed element (**a**) casing of the outer sleeve of elastic connector) (**b**). Photos of cross-sections of samples made in accordance with the horizontal (**c**) and vertical (**d**) orientations. 10:1 scale photograph of this ABS material structure deformed (**e**) and idealized model of this structure (**f**).

To check issues of the orthotropic structure in order to implement a material model of 3D-printed elements, the plane **ab** compatible with the print plane was defined (Figure 9a). To determine material parameters, a static tensile test and static torsion test of cylindrical samples were conducted. The samples were made in two orientations with respect to the main plane of the **ab** print: the horizontal orientation and vertical orientation. The3D-printed elements were prepared using the 90% filling density setting with the pattern given in Figure 9b. The parameters of the 3D printer (device name: Zortrax M200, Zortrax, Olsztyn, Poland) used to print elements were as follows: nozzle diameter 0.4 mm and layer height 0.19 mm. In order to identify the structure of the material, two types of samples were prepared: one in the horizontal direction and the second one in the vertical direction (Figure 9c,d).

**Figure 10:**
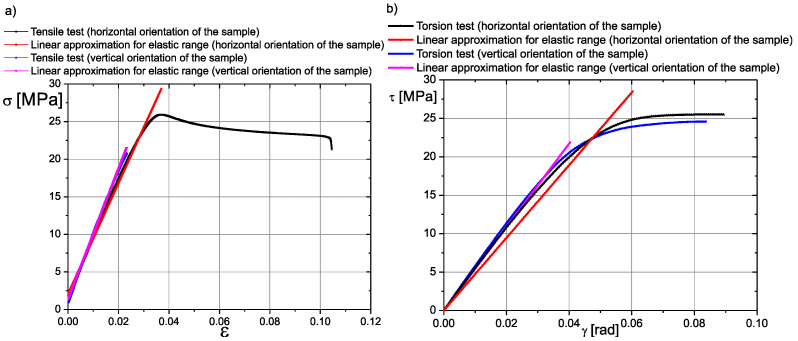
Results of tensile static test (**a**) and torsion static test (**b**) for ABS material.

The averaged results of the tensile static test and torsion static test of the 3D-printed samples are given in Figure 10. Linear approximations of the data were performed only for the elastic range of σ-ε and τ-γ.

Considering experimental results of the tensile static test and torsion static test, the following parameters were assessed (Section S4, Equation (S5)):

(1) Directional Young’s modulus *E_a_*; Poisson’s ratios *ν_ab_* and *ν_ac_*; tensile strength *R_ma_* and shear modulus *G_ab_*, *G_ac_* for the horizontal orientation material; 

(2) Directional Young’s modulus *E_b_* and *E_c_*; Poisson’s ratio *ν_bc_*; tensile strength *R_mb_* and *R_mc_* and shear modulus *G_bc_* for the vertical orientation material.

Averaged values of the mechanical properties are given in Table S1 (Section S5).

Analyzing the tensile static test results (Figure 10a), we assumed that the range of proportionality does not exceed 4% of the strain. We also assumed that the material behaves in symmetrical way (tension–compression). This assumption was implemented by using formulas [33,34] (Section S4).

**Figure 11:**
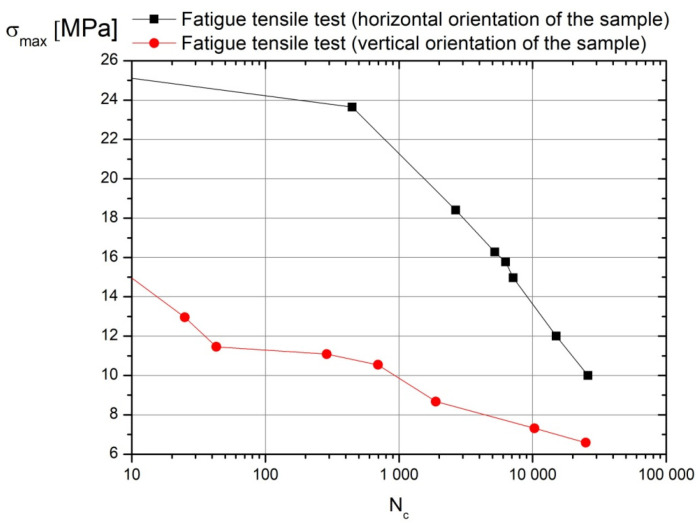
Wöhler curves for fatigue tensile test over one-sided repeated cycle for samples shown in Figure 9 (ABS material) (*σ_max_*—maximum tensile strength, *N_c_*—number of cycles).

Comparing the values of material parameters for the tested sample orientations, it is possible to notice a 14% difference in Young’s modulus and only a 6% difference in the shear modulus. However, there is a 23% difference in tensile strength. Therefore, the question arises whether this situation is sufficient for implementing an isotropic specific material model in FE models. To answer this question, it was decided to look at the structure of the specimen subjected to permanent deformation. For this purpose, the sample was subjected to shear loads (technical shear). A shearing tool was operated in the print planes, i.e., **ab**, **ac** and **bc**. In the results of this procedure, a fragment of the structure of the tested material is exposed. A 10:1 scale photograph of this structure is shown in Figure 9e. The idealized model of this structure is presented in Figure 9f. According to the presented drawings, printed components have an orthotropic structure, which becomes visible under load conditions. Therefore, the authors concluded that fatigue tests should be performed for samples subjected to tension in a one-sided repeated cycle. Samples prepared as described above were installed in a Zwick LTM10/Z010TE fatigue testing machine (Zwick Roell, Ulm, Germany). The load variation frequency was set to 0.5 Hz. The results of fatigue tests are presented in the form of Wöhler curves (Figure 11).

Taking into account the differences between the stress amplitudes presented on the Wöhler curves in relation to durability, it can be concluded that in FE models of the exoskeleton, a material model should be defined with parameters defining the constitutive law, taking into account orthotropy. In addition, this is supported by the fact that parts of the exoskeleton will be subjected to periodically varying loads with low frequencies. Thus, the results of the fatigue tests provided answers to the question of whether an orthotropic model can be used to simulate the stress state of exoskeleton parts made by the 3D printing technique.

### 2.3. Numerical Research

Numerical research of one side of the exoskeleton prototype was conducted by separately applying static loading to three discretized assemblies: (1) an ankle joint assembly composed of 658,417 3D-solid finite elements (Section S5); (2) a knee joint assembly composed of 340,202 3D-solid finite elements (Section S6) and (3) a hip joint assembly composed of 3D-220,550 solid finite elements (Section S7) (Figure 6, Figure 7 and Figure 8). A detailed description of the mesh models (Sections S5–S7) and properties of the used materials are given (Tables S1–S3). The number of finite elements of the model was determined based on a study of the sensitivity of the meshes in relation to the obtained values of effective stresses. A description of these tests is presented in Section S4.

The core of each screw was modeled by two-node beam elements (Section S5—Figure S.5.2). Each spring connector was modeled by two node rod elements (Section S6—Figures S.6.1 and S.6.3) [33,34].

Numerical research was performed as quasi-fatigue analysis, i.e., each step of the FEM model was statically loaded. The initial configuration of the models was carried out over two steps: (1) a subject and exoskeleton prototype weight (2*F_p_* = 900 N) was applied to the left foot during balancing on one side in the frontal plane of the body by setting zero angles *α* = *β* = *δ* = 0° (Figure 4, Figure 6 and Figure 7) (called State 0 in FEM analysis); (2) nominal torque was applied at chosen driving motors in the following way: first, in the heel-off phase, torque *T_1L_*= 40 Nm (Figure 2 and Figure S.5.3) was inputted (called State 1 in FEM analysis); second, torque *T_2L_* = 40 Nm (Figure 2 and Figure S.5.3) was inputted (called State 2 in FEM analysis), and it evoked an increase in angle *β*; third, an angular position between a heel segment and flat ground surface was inclined at *α* = 10°, *β* = 5° (according to [24]) by maintaining the straightened knee joint assembly *δ* = 0° (called State 3 in FEM analysis). 

In the next step, results of internal reactions originating in nodes sharing a common plane between the knee joint assembly and hip joint assembly were applied to the hip joint assembly as boundary conditions and input load.

## 3. Results

### 3.1. Experimental Research

**Figure 12:**
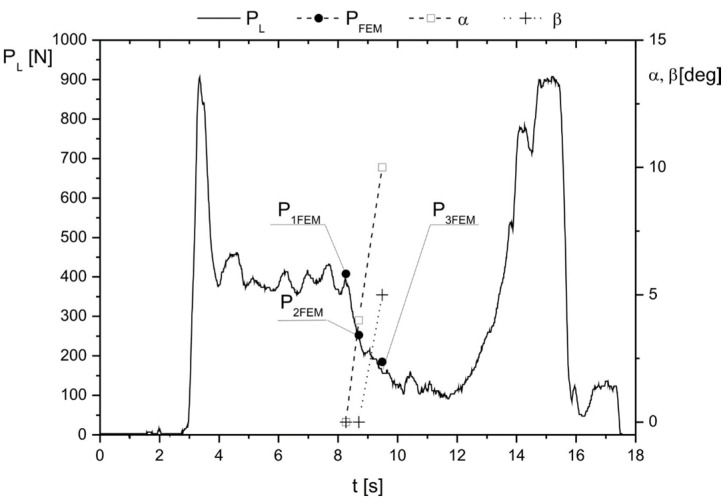
Time series of ground reaction *P_L_* measured under the subject’s left foot during balancing.

To validate the FEM models used to perform the numerical study (Section 2.3), chosen values of the resultant vertical reaction occurring between a ground surface EGI-25 (Figure S5.1) and sharing plane of the outer sleeve (3) during balancing (*P_1FEM_*= 408 N (State 1), *P_2FEM_* = 253 N (State 2), *P_3FEM_* = 185 N (State 3) given in Figures S2.1 and S.5.3 ) were compared with experimental ones measured by a force plate (Figure 12). Considering this time series, specific points and intervals were defined:

(1) at 3 s, the ground reaction force under the left foot equaled the weight of the exoskeleton and subject (2*F_p_* = 900 N) (State 0);

(2) over [3; 8.1) s, the pressure diminished due to balancing to the right side;

(3) over [8.1; 8.2) s, the angle *α* increased due to feet motion;

(4) at 8.2 s, a specific first state was defined (State 1);

(5) at 8.6 s, a specific second state was defined (State 2);

(6) over (8.6; 9.5)s, the feet motion occurred due to the simultaneous increase in angle *α* (to 10°) and angle *β* (from 0° to 5°);

(7) at 9.5 s, a specific third state was defined (State 3).

**Figure 13:**
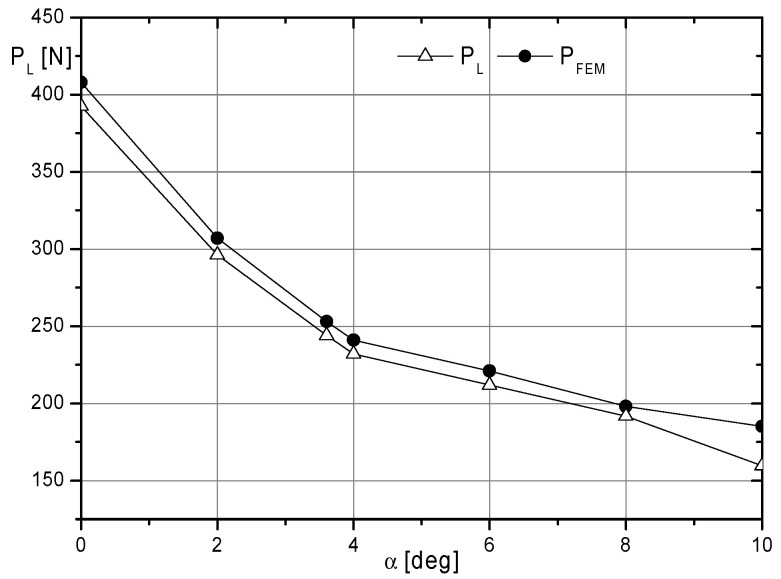
Summary of data for validation of the MES model (reactions obtained during measurement *P_L_* and results of FEM calculations *P_FEM_* as a function of angle *α.*

It should be noted that the validation of the FEM model included only the time interval in which the aforementioned moments caused an increase in the angles α and β (Figure 11). Taking into account the static analysis of the model to illustrate validation results, additional reaction values *P_FEM_* were determined. The values of these reactions (P_FEM_) were compared with the reaction values obtained from measurements (P_L_) in relation to angle α (Figure 13). 

Numerical evaluation of the model validation based on the presented data was performed using mean squared deviations *P_dev_* according to the formula:

(1) $$P_{dev}=\sqrt{\frac{\sum_{n}\left({P_{FEM}}^{2}-{P_{L}}^{2}\right)}{n}}$$

where:

*P_FEM_*—reaction calculated according to the FEM model at angle α;

*P_L_*—reaction measured at angle α;

*n*—number of data considered.

The mean squared deviation value for the received data is *P_dev_*= 13.5 N. This value, in relation to the range of measured reactions, indicates that the model results may be overestimated from 3 to 8%.

### 3.2. Results of Numerical Testing of Chosen 3D-Printed Elements

Based on FEM research, stress fields were determined in assembly elements (Sections S4–S7 and Tables S1–S3). For each element made from isotropic material, the yielding strength (Tables S1–S3) was treated as a strength limit. This limit was exceeded in the following elements: EGI-14 (Section S5); EGII-4 and EGII-9 (Section S6); EGIII-1, EGIII-2, EGIII-3 and EGIII-4 (Section S7).

**Figure 14:**
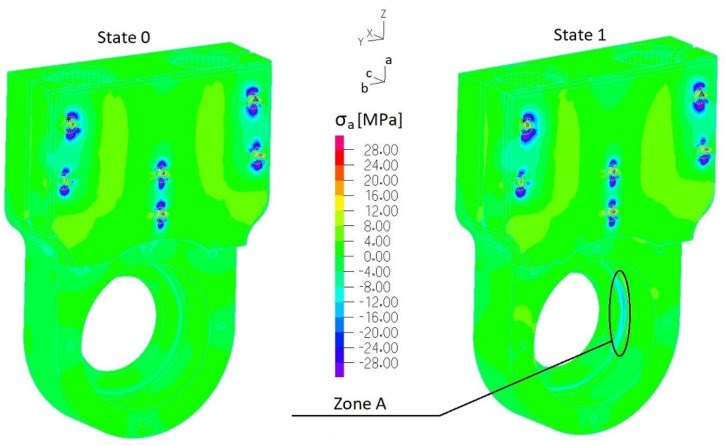
Distribution of normal stresses σ*_a_* in the casing of the outer sleeve of the elastic connector (EGI-4 (Section S5)) (State 0 and State 1).

**Figure 15:**
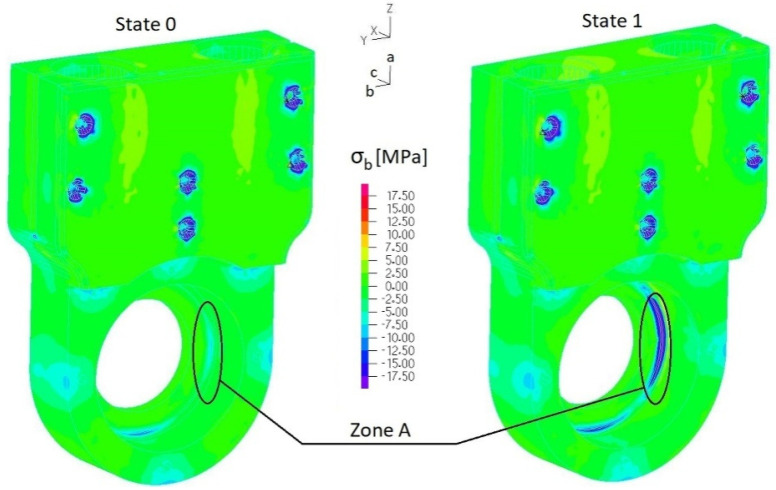
Distribution of normal stresses σ*_b_* in the casing of the outer sleeve of the elastic connector (EGI-4 (Section S5)) (State 0 and State 1).

Stress fields of the 3D-printed element (ABS) were analyzed along three orthogonal directions *σ_a_*, *σ_b_* and *σ_c_* (Figure 9) by considering the following tensile strength: *R_ma_* = 26 MPa and *R_mb_* = *R_mc_* = 20 MPa. Considering the most loaded elements of considered assemblies, the following parts were selected: the casing of the outer sleeve of the elastic connector (finite element group EGI-4) (Figure 4a), regulation clamp *h_8_* (finite element group EGI-12) (Figure 4a) and the lower housing of the internal sleeve of the elastic connector (finite element group EGII-9) (Figure 4b). Normal stress distributions along each orthogonal plane are presented in Figure 12, Figure 13, Figure 14 and Figure 15 and Figures S.8.1–S.8.6.

**Figure 16:**
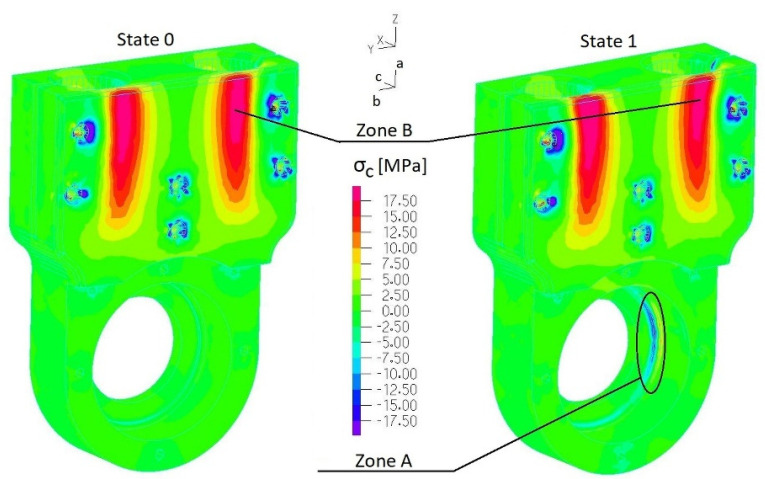
Distribution of normal stresses σ*_c_* in the casing of the outer sleeve of the elastic connector (EGI-4 (Section S5)) (State 0 and State 1).

**Figure 17:**
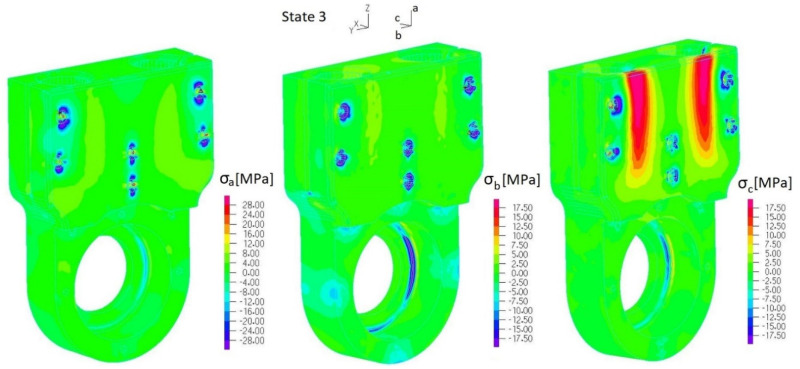
Distribution of normal stresses *σ_a_*, *σ_b_* and *σ_c_* in the casing of the outer sleeve of the elastic connector (EGI-4 (Section S5)) (State 3).

Figure 14, Figure 15, Figure 16 and Figure 17 present distributions of normal stresses *σ_a_*, *σ_b_* and *σ_c_* in the casing of the outer sleeve of the elastic connector (EGI-4) along orthogonal directions in State 0, State 1 and State 3. Analyzing these results, we found that (1) stress concentrations occurred in the screw hole, but normal stress values did not exceed the tensile strength; (2) the maximum stress is in ***zone A*** (Figure 15 and Figure 17): *σ_bmin_* = −18 MPa (State 1) and *σ_bmax_* = −14 MPa (State 3) (with quasi-static amplitude Δ*σ_a_* = 2 MPa). Also, we found that maximum stress *σ_c_* in ***zone B*** (Figure 16) exceeded the tensile strength in State 0. This indicates that this element cannot be used to transfer load in a permanent way. 

## 4. Discussion and Conclusions

Considering the results of quasi-fatigue load, one can relate them to the Wöhler curves that were identified in experimental fatigue tests (Figure 11). These curves were identified for the fatigue tensile test over a one-sided repeated cycle (0.5 Hz) for samples shown in Figure 9 (ABS material). This relation is based on two principal assumptions: (1) the exoskeleton should help perform slow motions (without acceleration); (2) exoskeleton elements are supposed to transmit quasi-fatigue loading due to the changing of the exoskeleton configuration and position of the body with respect to the exoskeleton (displacement of the center of mass during balancing, stepping and gait).

Results of the stress distributions for the tested assemblies (Sections S5–S7) were compared with the experimentally identified tensile strength values (*R_ma_*, *R_mb_* and *R_mc_*). This allowed stress concentration areas in 3D-printed elements to be identified. Moreover, to predict a durability of 3D-printed elements over given time, one can use the Wöhler curves (Figure 11).

We found that the difference between the tensile strength of horizontally oriented samples with respect to the vertically oriented one is 23% (Figure 9a). However, in the case of fatigue loads (Wöhler curves), the difference between these two differently oriented samples is 30%. Assuming that a 3D-printed element carried 10,000 cycles, this difference is increased to more than 50% (Figure 11). On the basis of our study, we state that material orthotropy has a substantial impact on the durability of load-bearing 3D-printed elements. That is why material properties should be carefully identified in practice and modeled by using the FEM approach. 

Focusing on FEM implementation, beam elements used to model initial stress corresponding to screw preload force *F_B_* (Tables S1–S3) can also be applied to solve an optimization task concerning the minimization of quasi-static stress amplitude in clench elements. Reducing this stress amplitude is very important in increasing the durability of load-bearing elements under fatigue loading conditions.

There are two main limitations in the presented exoskeleton FE model. The first concerns the inability to perform a modal analysis. The model did not define initial conditions, and the FEM analysis is a static analysis (representing the momentary state of dynamic load over time. The second limitation concerned the constitutive law. It was assumed that the structures of the modeled objects would be analyzed in the range of small deformations, which was implemented using Equations (S4)–(S9).

The presented model, compared to the models in [24,25,26], allows the implementation of parameters representing orthotropic materials. Taking into account contact conditions and introducing beam elements representing bolts ensures better compatibility of the model with the real system. Dividing the structure of the exoskeleton model into individual assemblies allows the use of higher-density meshes. Such a procedure also ensures greater accuracy of solutions and faster numerical convergence finding in solving contact issues between cooperating elements. Moreover, such partial models can facilitate the process of optimizing the shapes and dimensions of parts depending on defined loads.

## 5. Summary

The developed models of individual exoskeleton prototype workgroups enabled the selection of parts that meet the strength requirements. The experimental tests of the ABS material (static test, fatigue test), as well as the tests checking the correctness of the boundary conditions of the FEM model (Section 3.1), were proved to be necessary. The results of the stress distribution obtained from the FEM calculations and the comparison with the limit values *R_ma_*, *R_mb_* and *R_mc_* allowed one to indicate the geometrical regions of the part (made in the FDM technology) at which the dimensions should be increased. On the other hand, taking into account the results of fatigue tests, it is possible to predict in which areas the failure may be initiated. Knowledge of stress variability made it possible to develop a prognosis of the durability of individual components measured with the number of cycles. Thus, it can be determined whether the designed component can be safely used during the testing phase of the device. For this purpose, a series of fatigue tests was carried out to determine the Wöhler curves.

The research focused on the orthogonal orientation due to the orthogonality of the shapes of the considered exoskeleton components and the conditions of numerical adjustment of the experimentally determined data of the material model implemented in the ADINA system.

The values of material matrix components (Formula (S5)) given in the paper can be entered into libraries of commercial computing systems such as SolidWorks and ANSYS.

The analysis showed that it is possible that the optimal value of the preload force of the *F_B_* bolts (Section S5) used in the clamping connections can be derived. As a result, minimization of amplitudes of variable stresses can be obtained in the combined parts, which is a desirable phenomenon from the point of view of the fatigue strength of materials. The obtained displacement values of individual exoskeleton assemblies can be used to assess the impact of system stiffness on the patient’s comfort. The obtained reactions in the nodes of the model make it possible to determine the forces acting on the human limb. 

It is planned to extend the analysis based on the results of ongoing fatigue tests in the range of the contact strength of flat, concave and convex surfaces of the ABS material. Basic analyses of this issue, using the example of a gear transmission (Figure S.3.1), are presented in the publication [35].

The stresses in the components of the prototype made using the FDM technique, compared with the limit states of the tested material (ABS), gave information that these elements would not be able to meet the strength requirements for the maximum loads of the exoskeleton. Both material samples and components were made without any additional technological treatment. Therefore, it is necessary to consider the current achievements that allow an increase in the value of the limit state parameters of the ABS material, e.g., by heating the manufactured components, which was presented in the publication [9]. Referring to the graphs presented there describing the static and fatigue strength when this technology is used, the designed components of the exoskeleton should achieve all strength requirements. Hence, the developed finite element model structure can be reused to simulate the loads. 

On the basis of the presented description of the numerical model in terms of large displacements, large rotations and small deformations, it is possible to easily build models of similar objects, but special attention should be paid to determining the parameters of the constitutive law of the material, which was confirmed in the results of the experiment.

## Data Availability

The original contributions presented in the study are included in the article/supplementary material, further inquiries can be directed to the corresponding author.

## Supplementary Materials

The following supporting information can be downloaded at: https://www.mdpi.com/article/10.3390/ma17143406/s1. References [36,37,38,39,40] are cited in the supplementary materials.
