# Supplementary material for: Modeling and Strength Calculations of Parts Made Using 3D Printing Technology and Mounted in a Custom-Made Lower Limb Exoskeleton
Source: Materials (Basel). 2024 Jul 10;17(14):3406. doi: 10.3390/ma17143406 (PMC11278006; doi:10.3390/ma17143406)
Supplement: Supplementary file 1 [file materials-17-03406-s001.zip › materials-3043867-supplementary.pdf]

Supplementary Materials:

S.1. Abbreviations
S.2. Elastic connector
S.3. The driving unit
S.4. FEM analysis description
S.5 FEM model of the ankle joint assembly
S.6 FEM model of the knee joint assembly
S.7 FEM model of the hip joint assembly
S.8 FEM results of chosen 3D printed elements

Supplementary

S1. Abbreviations

S_R [N] — weight of the right vertical exoskeleton module (Fig. 2)

S_L [N] — weight of the left vertical exoskeleton module (Fig. 2)

S_C[N] — weight of the central horizontal exoskeleton module (Fig. 2)

G_R [N] — a resultant of pressure forces coming from the right foot of the patient (Fig. 2)

G_L [N] — a resultant of pressure forces coming from the left foot of the patient (Fig. 2)

i [-] — gear ratio of the conical gears (Fig. S.3.1)

m [mm] — transverse module of the conical gears (Fig. S.3.1)

α [deg] — angle between the heel segment and the ground measured in the yz sagittal plane (Fig. 6)

β [deg] — angle between the heel segment and the ground measured in the xz frontal plane (Fig. 6)

α_n [deg] — nominal pressure angle of the conical gear wheel (Fig. S.3.1)

β_n [deg] — helix angle of the conical gear wheel (Fig. S.3.1)

μ [-] — sliding friction coefficient used in the FEM model

ε [-] — strain

σ [MPa] — stress

σ_1 [MPa] — limit stress of a truss element in the FEM model (Fig. **S.6.2**)

σ_0 [MPa] — effective stress

τ[MPa]—torsional stress (Fig. 10b)

γ[rad] —angle of shear deformation (Fig. 10b)

σ_a, σ_b, σ_c [MPa] — components of normal stress in orthogonal directions *abc* for orthotropic material

σ_{amin}, σ_{bmin}, σ_{cmin} [MPa] — minimal normal stress in orthogonal directions *abc* for orthotropic material

σ_{amax}, σ_{bmax}, σ_{cmax} [MPa] — maximal normal stress in orthogonal directions *abc* for orthotropic material

$\Delta\sigma_a$, $\Delta\sigma_b$, $\Delta\sigma_c$ [MPa] — amplitudes of time variable normal stress in orthogonal directions *abc* for orthotropic material

ε_1 [-] — limit strain of a truss element in the FEM model (Fig. S.6.2)

E_a,E_b, E_c [MPa] — Young's modulus of the orthotropic material model (Tables S1, S2, S3) according to planes (Fig. 9)

E [MPa] — Young's modulus of the isotropic material model (Tables S1, S2, S3)

E_1, E_2 [MPa] — modulus of the bilinear material characteristic (Fig. S.6.2)

ν[-] — Poisson's ratio of the isotropic material model (Tables S1, S2, S3)

G_{ba}, G_{cb}, G_{ca} [MPa] — shear modulus of the orthotropic material model (Tables S1, S2, S3) according to planes (Fig. 9)

ν_{ba}, ν_{cb}, ν_{ca} [-] — Poisson's ratio of the orthotropic material model (Tables S1, S2, S3) by plane (Fig. 9)

R_{ma}, R_{mb}, R_{mc} [MPa] — minimal strength of orthotropic material in *abc* directions of material structure (Fig. 9)

z — number of teeth for both wheels of the conical gear (Fig. S.3.1)

d_m [mm] — mean pitch diameter of the conical gear (Fig. S.3.1)

θ_1, θ_2 — semiangle of the pitch conic in the active and passive conical gear wheel

M_n [Nm] — torque transmitted between the conical gear wheels (Fig. S.3.1)

F_o [N] — circumferential tooth force in the conical gear (Fig. S.3.1)

F_r [N] — radial tooth force of the active wheel in the conical gear (Fig. S.3.1)

F_a [N] — axial tooth force of the active wheel in the conical gear (Fig. S.3.1)

d_3 [mm] — minor diameter of the screw used in exoskeleton modules (Tables S1, S2, S3)

F_p [N] — the resultant normal force between foot and the heel module (Fig. S.5.3)

F_B [N] — preload force of a screw in FEM model (Tables S1, S2, S3)

T_{1y}, T_{1z}, T_{2x}, T_{2z}, T_{3x}, T_{3y}, T_{4y}, T_{4z} [Nm] — bending moments acting on the components of the propulsion system (Fig. S.5.3)

T_{1x}, T_{2y}, T_{3z}, T_{4x} [Nm] — torsional moments acting on the components of the propulsion system (Fig. S.5.3)

δ [deg] — knee joint angle (Fig.7)

A [m^2] — cross-sectional area of a truss-type finite element (Table S2)

L [m] — length of truss-type finite element (Table S2)

k [N/mm] — linear spring constant (Table S2)

ΔL [mm] — adjustment length of the elastic connector (Fig. S.6.2)

F_{Fx}, F_{Fy}, F_{Bx}, F_{By}, F_{Fz}, F_{Bz} [N] — external load components introduced into the knee joint model (Fig. S.6.3)

F_x, F_y, F_z [N] — external load components introduced into the hip joint model (Fig. S.7.2)

e_1, e_2, e_3 [m] — dimensions determining the points of application of forces F_x, F_y, F_z in the hip joint model (Fig. **S.7.2**)

P_L [N] — ground reaction force under the patient's left foot

P_{dev} [N] — mean squared deviations

P_{FEM}—reaction calculated according to the FEM model

S2. Elastic connector

The axial displacement between assemblies I-II and II-III (Fig.1) is carried out by a guideblock (4) in the range of the maximum stroke of the spring (1) (subjected to compression-tension force along an adjustment range ΔL). This spring is embedded in the internal sleeve (2) and can move slidably in the outer sleeve (3). To adjust parameters

h_1 and h_2 (Fig. 2), the mandrel (5) is used. In set position, this mandrel is attached to the outer sleeve (3).

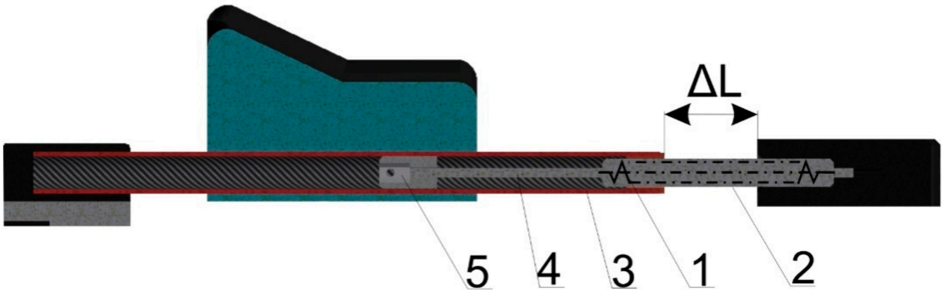

Figure S.2.1. Construction of the elastic connectors. Part names: spring (1), internal sleeve (2), outer sleeve (3), guideblock (4), mandrel (5).

S3. The driving unit

The driving unit is composed of a stepper motor (1) coupled with the planetary gear (2) that is connected to a conical gear in which the axles of the driving wheel (4) and the driven wheel (5) are perpendicular. The driven wheel is coupled to the shaft (15 in Fig. 4b) by a machine key. The shaft is mounted in a rolling bearing (6). The motor (1), together with the gear (2), is attached to the engine mount (3).

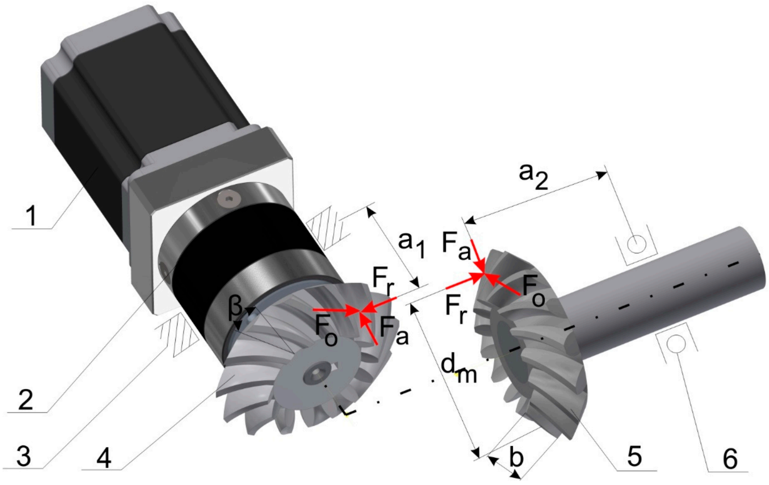

Figure S.3.1 Construction and load scheme of the exoskeleton driving unit. Part names: stepper motor (1), planetary gear (2), engine mount (3), driving wheel (4) and the driven wheel (5), rolling bearing (6).

Due to significant unit load transmission by the teeth of the conical gear, curvilinear teeth were used. The basic parameters of the conical gear are: gear ratio i= 1, transverse module m=5 mm, tooth trace b = 20 mm, nominal pressure angle α_n = 20°, helix angle of tooth β_n = 34°, the number of teeth z=14, mean pitch diameter d_m = 56 mm, semi-angle of the pitch conic of the driving wheel θ_1 = 45°, and driven wheel θ_2 = 45°. The torque M_n is transmitted between the gears of the conical gear generates loads acting on the shafts.

The main components of these loads are designed on the basis of the value of contact forces located between the teeth, acting in orthogonal directions, i.e., the circumferential force of the driving wheel F_o, the radial force of the driving wheel F_r, and axial force of the

driving wheel F_a. The values of these forces can be determined by the following formulas:

$$F_o = \frac{2M_n}{d_m} \tag{S1}$$

$$F_a = F_o \cdot \left(\frac{tg\alpha_n \cdot sin\theta_1}{cos\beta_n} + tg\beta_n \cdot cos\theta_1\right) \tag{S2}$$

$$F_r = F_o \cdot \left(\frac{tg\alpha_n \cdot sin\theta_2}{cos\beta_n} - tg\beta_n \cdot cos\theta_2\right) \tag{S3}$$

Using the values of contact forces (circumferential force of the driving wheel F_o, radial force of the driving wheel F_r, and axial force of the driving wheel F_a), one can define clustered moments (11) – (14). It is worth noticing that contact forces (2) – (4) evoke additional loadings (other than torque transmitted between the conical gear wheels M_n) that superimpose all exoskeleton elements (S.5, S.6).

S4. FEM analysis description

Based on the design assumptions of the exoskeleton use conditions, it can be concluded that the modeled parts will experience large displacements and large rotations. The linearization of material characteristics σ-ε obtained from strength tests (Fig. 10a), and in particular from the orthotropic properties of ABS material, allowed one to state that for all defined material models (3D-solid and beam-type finite elements), the range of the proportionality (linearity) is the strain ε = 4 %. Therefore, it was assumed that the structure of modeled objects would be subject to the law of small deformations. Based on this statement, the equations [33, 34] were selected and used in the ADINA system to formulate the definition of individual 3D-solid and beam-type finite elements, with the possibility of implementing all material parameters listed in Tables S1-S3.

In order to perform the FEM analysis of the exoskeleton load, it was necessary to define the variable describing successive states of positions and stresses, which is the so-called time increment Δt (time increment). The strategy for solving such a task assumes that the solution for static and kinematic variables will be obtained for all time steps. A typical solution is to obtain an equilibrium position of the object for time t + Δt. In incremental Lagrangian analysis, the equilibrium of the body for the step t + Δt is found using the principle of virtual displacements.

In reference to the material model according to Voigt's notation, a Hooke's law is derived by using a stiffness matrix C_{ij}. In the case of isotropic properties, this stiffness matrix has a form:

$$C_{ij} = \frac{E}{1+\nu}\begin{bmatrix} \frac{1-\nu}{1-2\nu} & \frac{\nu}{1-2\nu} & \frac{1-\nu}{1-2\nu} & 0 & 0 & 0 \\ & \frac{1-\nu}{1-2\nu} & \frac{1-\nu}{1-2\nu} & 0 & 0 & 0 \\ & & \frac{1-\nu}{1-2\nu} & 0 & 0 & 0 \\ & & & \frac{1}{2} & 0 & 0 \\ & \text{symmetric} & & & \frac{1}{2} & 0 \\ & & & & & \frac{1}{2} \end{bmatrix} \tag{S4}$$

In the case of orthotropic properties, this stiffness matrix Cij has the following form:

$$C_{ij} = \begin{bmatrix} \frac{1-\nu_{bc}\nu_{cb}}{E_bE_c\Delta} & \frac{\nu_{ba}+\nu_{bc}\nu_{ca}}{E_bE_c\Delta} & \frac{\nu_{ca}+\nu_{ba}\nu_{cb}}{E_bE_c\Delta} & 0 & 0 & 0 \\ & \frac{1-\nu_{ac}\nu_{ca}}{E_aE_c\Delta} & \frac{\nu_{ba}+\nu_{bc}\nu_{ca}}{E_bE_c\Delta} & 0 & 0 & 0 \\ & & \frac{1-\nu_{ab}\nu_{ba}}{E_aE_b\Delta} & 0 & 0 & 0 \\ & & & G_{bc} & 0 & 0 \\ & \text{symmetric} & & 0 & G_{ac} & 0 \\ & & & 0 & 0 & G_{ab} \end{bmatrix} \quad (S5)$$

where:

$$\Delta = \frac{1 - \nu_{ab}\nu_{ba} - \nu_{bc}\nu_{cb} - \nu_{ca}\nu_{ac} - 2\nu_{ba}\nu_{cb}\nu_{ac}}{E_aE_bE_c}$$

Moreover, components of the stiffness matrix (S5) should satisfy the following constraints:

$$E_i, G_{ij} > 0 \quad (S6)$$

$$|\nu_{ij}| < \left(\frac{E_i}{E_j}\right)^{\frac{1}{2}} \quad (S7)$$

wherei,j = a, b, c

$$\nu_{ba}\nu_{cb}\nu_{ac} < \frac{1-\nu_{ab}\nu_{ba}-\nu_{bc}\nu_{cb}-\nu_{ac}\nu_{ca}}{2} < \frac{1-\nu_{ba}^2\left(\frac{E_a}{E_b}\right)-\nu_{cb}^2\left(\frac{E_b}{E_c}\right)-\nu_{ac}^2\left(\frac{E_c}{E_a}\right)}{2} \quad (S8)$$

Modeling a rod element, which sustains only uniaxial load (resultant of nodal forces *F*), the FEM implementation is realized by considering formulas (S14)-(S16), and Fig. S.6.2. The maximum strain of the rod element is assessed by using the relation:

$$\varepsilon_{max}^{(t)} = \frac{\sigma_1}{E_1} + \frac{\frac{F_{max}}{A}}{E_2} \quad (S10)$$

FEM models of 3D printed parts consist of eight-nodal finite elements type 3D-Solid. The same type of element was used to discretize components made of materials with isotropic features (formula S5). The consequence of using FEM is the need to check the influence of the discretization density of the exoskeleton geometric objects on the calculation result. In the presented case, the number of finite elements per millimeter of the edge length of the discretized objects was determined as a measure of the discretization density.

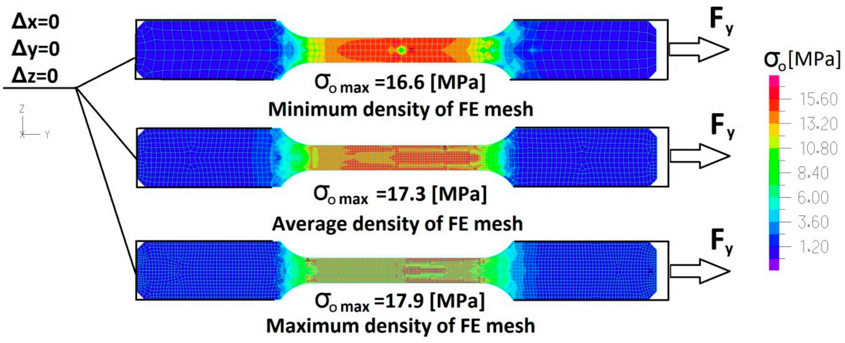

Figure S.4.1. Distributions of effective stress for FEM meshes in material sample models tested.

The mesh sensitivity test was carried out using the geometry of material samples subjected to strength tests. The sample models were conditioned according to the conditions of installation and loading of the samples in the testing machine. The material model is described by equation (S5). The models of samples were loaded with a force F_y (Fig. S.4.1). The limiting value of this force was set so that the yield strength was not exceeded for samples printed in the vertical orientation (Fig. 10a). FEA analysis was performed on three different

mesh densities described in Figure S.4.1 as: minimum (0.7 finite element/mm), average (1.7 finite element/mm) and maximum. (2.7 finite elements/mm). The criterion for evaluating the convergence of solutions was the maximum effective stress σ_{omax}. The highest value of this stress was obtained for the model with max mesh density. Due to the small differences in the range of solutions found between models with average and maximum mesh density, an average density mesh was used to discretize the exoskeleton model. Mesh sensitivity analysis was performed using the geometry of material samples due to the possibility of controlling the numerical solution based on measured data.

S5. FEM model of the ankle joint assembly

Considering the mesh model of ankle joint assembly (Fig. 5), 25 groups of finite elements were defined (Fig. S.5.1, Table S1). These groups represent the geometric structures of the EGI-1 first engine clamp, EGI-2 first engine mount, EGI-3 outer sleeve of the elastic connector, EGI-4 housing of the outer sleeve of the elastic connector, EGI-5 bearing, EGI-6 cover, connecting screws EGI-7, EGI-8 conical heads of screws, EGI-9 first drive shaft, EGI-10 another set of screws with EGI-11 their heads, EGI-12 regulation clamp h_8, EGI-13 pin, EGI-14 heel module connectors, EGI-15 second engine mount, EGI-16 heel module connector, EGI-17 second drive shaft, EGI-18 second bearing, EGI-19 second cover, EGI-20 next screws group and their EGI-21 heads, EGI-22 second pin, EGI-23 patient's foot, EGI-24 heel module, and EGI-25 ground.

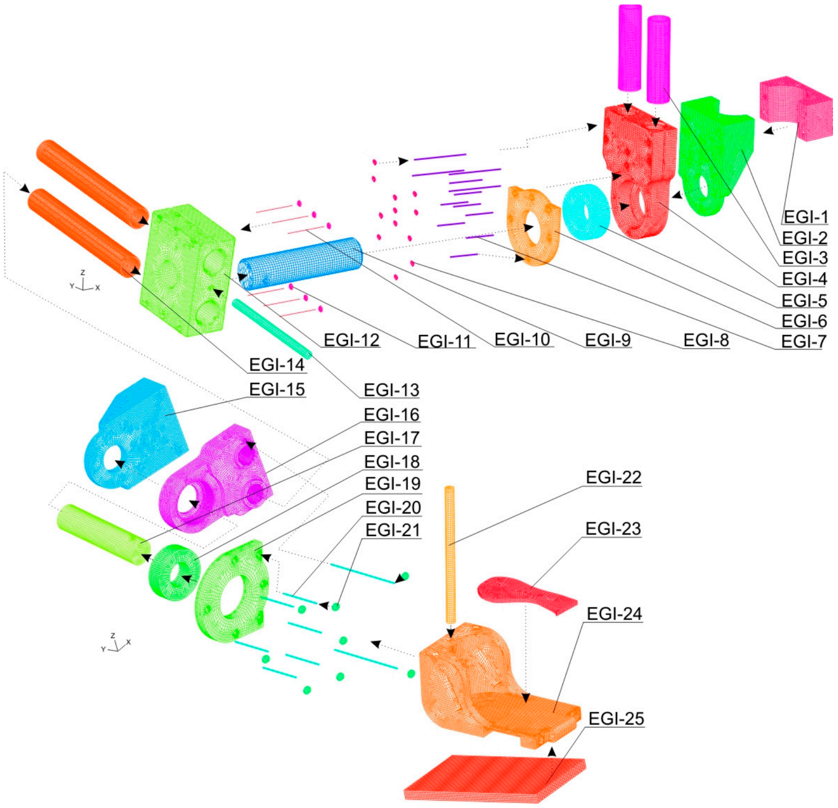

Figure S.5.1. Groups of finite elements of the ankle joint assembly.

Most of the groups are described by eight-node finite elements of the 3D-solid type [33, 34]. Exceptions are the groups representing the

following screws: EGI-7, EGI-10, and EGI-20. As in a previous study [36], each screw was modeled in a simplified manner using two node beam-type finite element by considering a material characteristic, length (to cover the rod of the screw), dimension of the cross-section, and the value of the preload force F_B [33, 34]. To determine an optimal value of this preload force, iterative calculations are performed by adapting beam elements (Fig. S.5.2). Each screw has a conical head, while its nuts have a hexagonal outline. To consider the interactions between the conical head surfaces and sockets, the surface contact conditions were defined by assuming penetration conditions and friction coefficient μ= 0.1 [33, 34, 37]. Contact conditions of this type have been introduced for pairs of common surfaces described by nodes of appropriate finite-element groups, i.e., EGI-1 and EGI-2, EGI-2 and EGI-4, EGI-3 and EGI-4, EGI-4 and EGI-5, EGI-4 and EGI-6, EGI-5 and EGI-6, EGI-4 and EGI-8, EGI-6 and EGI-8, EGI-11 and EGI-12, EGI-12 and EGI-14, EGI -12 and EGI-13, EGI-14 and EGI-16, EGI-9 and EGI-13, EGI-15 and EGI-16, EGI-16 and EGI-18, EGI-16 and EGI-19, EGI-16 and EGI-21, EGI-19 and EGI-21, EGI-22 and EGI-24, EGI-17 and EGI-22, EGI-24 and EGI-25. The coefficient of friction of the EGI-23 and EGI-24 groups depends on whether the patient's foot is covered. The distribution of nodes for the group EGI-23 was made by performing a laser scan of a patient’s foot (size 42 EUR was set). Displacements of the extreme nodes of all beam elements were determined by using ideally rigid links connected to all nodes located on the bottoms of the conical heads and the counter surfaces representing the nuts [33, 34]. It is worth noticing that this approach (implemented to model a screw connection) assumes that the shear stress is small and may be omitted [38].

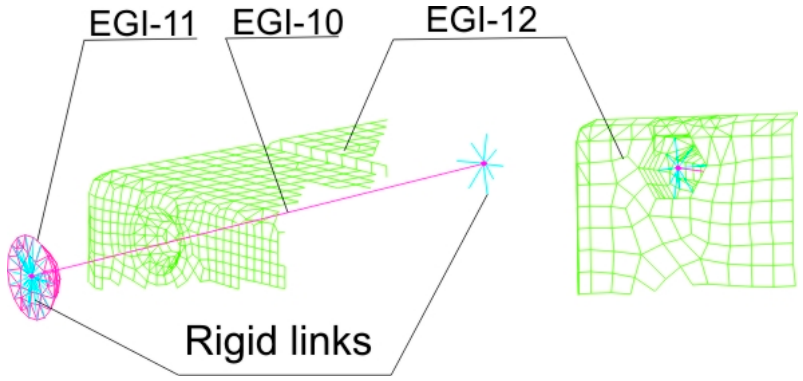

Figure S.5.2. Simplified modeling method for screw connections.

The rolling bearings in the model were replaced by rings (EGI-5 and EGI-18) with external dimensions equal to those of the mentioned parts. Between cylindrical surfaces, common to nodes from the EGI-5 and EGI-9, EGI-17 and EGI-28 groups, the assumed contact conditions were similar to those described above but considering an appropriate value for the rolling bearings friction coefficient μ= 0.0015 [39, 40].

The angles α and β shown in Fig. 6 are parametrically changed to take into account the internal loads of the assembly depending on the position of the patient's foot relative to the ground. The positions of nodes representing individual components of the assembly were parametrically related to angles α and β by introducing local coordinate systems. These systems are defined relative to the global

coordinate system by determining the coordinates of the starting point and Euler angles. The coordinates of the nodes from groups from EGI-9 to EGI-16 were assigned to the first local coordinate system, while finite nodes from EGI-17 to EGI-24 were assigned to the second coordinate system. The third local coordinate system is used for the nodes of EGI-25 group.

The material model parameters of all groups of finite elements of the ankle assembly model are summarized in Table S1. Numerical study was performed by separately testing: ankle joint assembly, knee joint assembly and hip joint assembly. This strategy was set to preserve internal forces in places where the interaction of two assemblies occurred. For this purpose, all the nodes of the ankle joint assembly located at the division site have been defined with zero displacements (EGI-3 group in Fig. S.5.3).

Table S1. Material model parameters of finite-element groups of the ankle joint assembly.

Figure	Part number	Finite-element group number (Fig. S.5.1 and S.5.2)	Material	Material model
4a	1	EGI-1	ABS	Orthotropic<br>E_a= 900 MPa, E_b = E_c= 1024 MPa<br>ν_{ab}= ν_{ac}=0.457, ν_{bc}= 0.520<br>G_{ab} = G_{ac}= 513 MPa, G_{bc}= 482 MPa<br>R_{am}=26 MPa, R_{bm}=20 MPa, R_{cm}=20 MPa
4a	2	EGI-2		
4a	3	EGI-4		
4a	5	EGI-12		
4a	7	EGI-24		
4a	9	EGI-16		
4a	2	EGI-15		
5b	5	EGI-6		
5b	5	EGI-19		
S.2.1	3	EGI-3	Polypropylene	Isotropic<br>E = 2000 MPa<br>ν= 0.3<br>R_e = 35 MPa
4a	6	EGI-14		
4b	12	EGII-14	Aluminum PA6	Isotropic<br>E= 70000 MPa<br>ν= 0.35<br>R_e = 240 MPa
4a	11	EGI-17		
4a	5	EGI-13	Steel	Isotropic<br>E= 200000 MPa<br>ν= 0.33
4a	10	EGI-22		
-	-	EGI-8		
-	-	EGI-11		
-	-	EGI-21		
4a	4	EGI-7	Steel	Isotropic<br>E= 200000 MPa<br>ν= 0.33<br>Circular cross section with the diameter of the screw core d_3 = 4 mm<br>R_e = 300 MPa<br>F_B= 1000 N
-	-	EGI-10		
-	-	EGI-20		
-	-	EGI-23	Compact tissue	Isotropic<br>E= 21500MPa<br>ν= 0.3

For this purpose, all the nodes of the ankle joint assembly located at the division site have been defined with zero displacement (EGI-3 group in Fig. S.5.3). The resultant reaction of these nodes was used as input loads in the knee joint assembly. Zero displacement values were defined for EGI-25 group nodes located on the bottom surface of the ground model. Other boundary conditions are caused by external loads acting on the assembly structure. The load corresponding to the foot resultant pressure F_P was applied at external nodes of the group EGI-23. The forces originated from a driving system (Fig. S.3.1) were replaced by the action of clustered moments:

$$T_{3y} = F_a \cdot \frac{d_m}{2} + F_r \cdot a_1 \tag{S10}$$

$$T_{3x} = F_p \cdot a_1 \tag{S11}$$

$$T_{1y} = F_r \cdot \frac{d_m}{2} + F_a \cdot a_2 \tag{S12}$$

$$T_{1z} = F_p \cdot a_2 \tag{S13}$$

These clustered moments were applied at additionally defined central nodes **CN1**, **CN2**, **CN3**, and **CN4** (Fig. S.5.3). The location of nodes **CN3** and **CN4** is derived from the center of gravity of the opening formed by the clamp and mount of the engine (Fig. 4a). The driven side is represented by **CN1** and **CN2** nodes located in the center of gravity of the driven gear. The interaction moments concentrated between central nodes and dependent nodes (representing the relevant parts of the assembly) were implemented by introducing rigid links [33, 34]. Nodes on the outer surface of the shaft (Fig. 4a) were connected and **CN2**. Due to the geometrical identity of the EGI-2 and EGI-15 groups, these moments were implemented by assuming: $M_n = T_{3z} = T_{1x} = T_{4x} = T_{2y}$, $T_{3y} = T_{4z}$, $T_{3x} = T_{4y}$, $T_{1y} = T_{2z}$, and $T_{1z} = T_{2x}$.

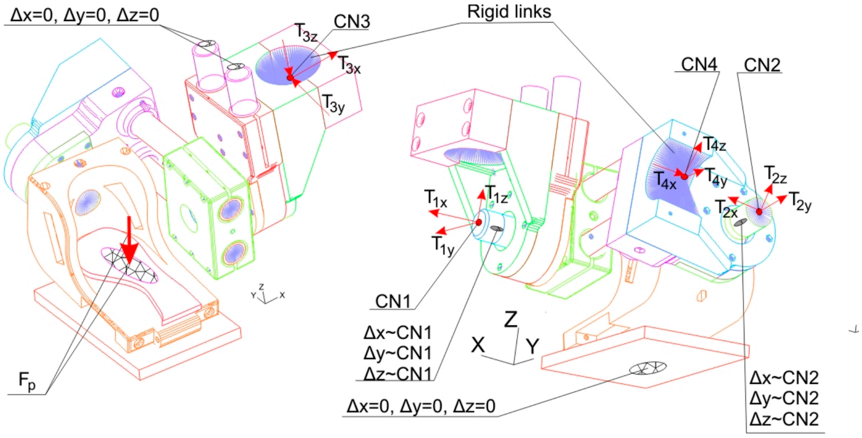

Figure S.5.3. Boundary conditions of the ankle joint assembly.

S6. FEM model of the knee joint assembly

Considering the mesh model of knee joint assembly (Fig. 7), 17 groups of finite elements were defined (Fig. S.6.1, Table S2). These

groups represent the geometric structure of EGII-1 engine clamp, EGII-2 engine mount, EGII-3 inner sleeve of the elastic connector, EGII-4 upper housing of inner sleeve of the elastic connector, EGII-5 bearing, EGII-6 cover, EGII-7 screws, EGII-8 conical heads of screws, EGII-9 lower housing of inner sleeve of the elastic connector, EGII-10 lower inner sleeve of the elastic connector, EGII-11 hex nuts, EGII-12 pins, EGII-13 second set of screws and EGII-15 their conical heads, EGI-14 the drive shaft, and spring of upper elastic connector EGII-16 and lower EGII-17.

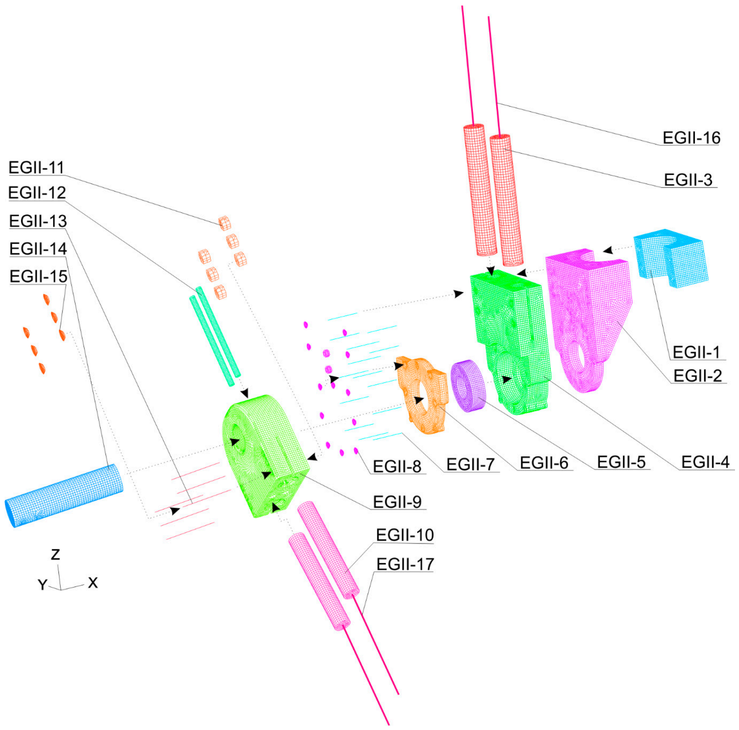

Figure S.6.1. Groups of finite elements of the knee joint assembly.

The boundary conditions of surface contact, including penetration and friction [33, 34, 37], are defined by the coefficient of friction μ= 0.1 for pairs of common surfaces described by nodes of appropriate finite-element groups, i.e., EGII-1 and EGII-2, EGII-2 and EGII-4, EGII-3 and EGII-4, EGII-4 and EGII-5, EGII-4 and EGII-6, EGII-4 and EGII-8, EGII-6 and EGII-8, EGII-9 and EGI-14, EGII-12 and EGII-14, EGII-9 and EGII-12, EGII-9 and EGII-10.

A bearing was replaced by a ring represented by group EGII-5, with the outer diameter equal to the actual part. Between cylindrical surfaces, common to nodes from groups EGII-5 and EGII-14, contact conditions were defined as previously with the friction coefficient μ= 0.0015 [39, 40].

A "glue" boundary condition was implemented on the common surface formed by the EGII-9 and EGII-11 nodes [33, 34]. These boundary conditions ensure unchanged relative positions of the nodes of different groups of finite element.

An angle δ shown in Fig. 7 was determined by using the parametrical approach to reflect the influence of internal loads depending on the position of the shin segment and thigh segment. The positions of the nodes representing the individual components of the assembly were parametrically associated with the angles α and β (Fig. 6) by entering the local coordinate systems described by the

coordinates of the starting point and the Euler angles. The coordinates of nodes of groups EGII-9 to EGII-15 and EGII-17 were assigned to the local coordinate system.

Most of the finite element groups of knee joint assembly were described by eight-node finite elements of the 3D-solid type [33, 34]. The first exception were groups representing screws: EGII-7 and EGII-13. As in the model of the ankle joint assembly, bolts were modeled as two-node beam-type elements [33, 34]. The way of adapting the beam elements to the model was the same as that used for the ankle joint assembly. The second exception was springs, which were modeled with two-node truss-type rod elements by considering that cross-section area A was constant. Considering influence of the elastic connector (S.2), the material model of the rod element was implemented in the form given in Fig. S.6.2.

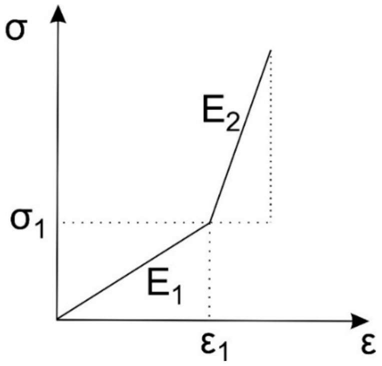

Figure S.6.2. Two-linear elastic material characteristic.

The longitudinal elastic modulus E_1 (Fig. S.6.2) is defined as follows:

$$E_1 = \frac{\sigma_1}{\varepsilon_1} \quad \text{(S14)}$$

where

$$\varepsilon_1 = \frac{\Delta L}{L} \quad \text{(S15)}$$

$$\sigma_1 = \frac{k \cdot \Delta L}{A} \quad \text{(S16)}$$

The elastic modulus E_2 is equal to Young's modulus of the finite-element groups EGII-3 and EGII-9.

Table S2. Material model parameters of finite-element groups of the knee assembly.

Figure	Part number	Group of finite elements (Fig. S.6.1.)	Material	Material model
4a	1	EGII-1	ABS	Orthotropic<br>E_a= 900 MPa, E_b = E_c= 1024 MPa<br>ν_{ab}= ν_{ac}=0.457, ν_{bc}= 0.520<br>G_{ab} = G_{ac}= 513 MPa,G_{bc}= 482 MPa<br>R_{am}=26 MPa, R_{bm}=20 MPa, R_{cm}=20 MPa
4a	2	EGII-2		
4b	14	EGII-4		
6b	23	EGII-6		
4b	16	EGII-9		
4a	9	EGI-16		
4a	2	EGI-15		
5b	5	EGI-6		
5b	5	EGI-19		
4b	15	EGI-14	Aluminum PA6	Isotropic<br>E = 70000 MPa<br>ν = 0.35

				R_e=240 MPa
S.2.1	2	EGII-3	Steel	Isotropic<br>E = 200000 MPa<br>ν= 0.33<br>R_e = 300 MPa
4a	10	EGII-5		
-	-	EGII-8		
S.2.1	2	EGII-10		
-	-	EGII-11		
-	-	EGII-12		
-	-	EGII-15		
4a	4	EGI-7	Steel	Isotropic<br>E= 200000 MPa<br>ν = 0.33<br>Circular crosssection with the diameter of the screw core<br>d_3 = 4 mm<br>F_B = 1000 N
-	-	EGI-13		
S.2.1	1	EGII-16	Spring steel	Two-linear elastic<br>k= 1 N/mm<br>ΔL= 20 mm<br>L= 100 mm<br>A = 0.00011304 m2<br>ε_1 = 0.2<br>σ_1 = 0.176928 MPa<br>E_2 = 200000 MPa

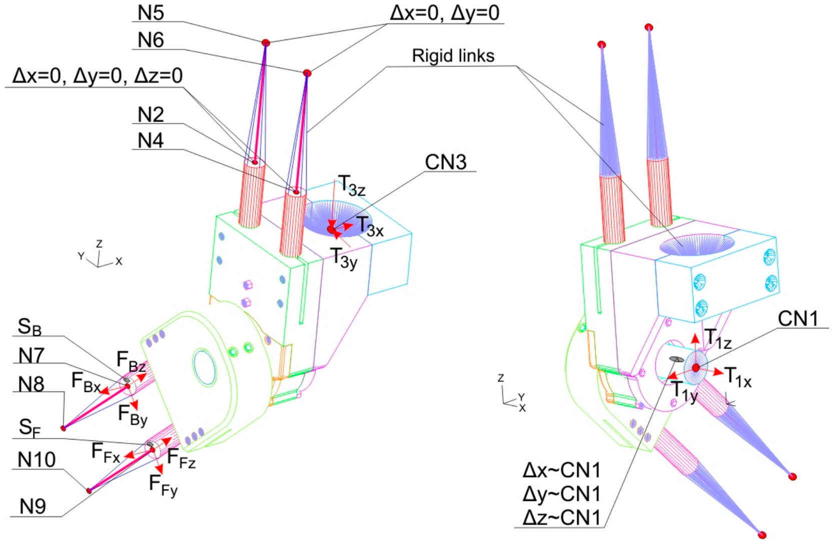

Figure S.6.3. Boundary conditions of the knee joint assembly.

The rod elements that were introduced into the model to correctly represent the action of the elastic connector required to apply appropriate boundary conditions caused by connections between nodes of neighboring finite element groups (Fig. S.6.3). The upper forward rod element was described by the extreme nodes **N4** and **N6**, and the upper rear rod element was defined by nodes **N2** and **N5**. To maintain the continuity of loads between the knee and hip joint assemblies, as well as to identify the internal load components based on the calculated reaction components in the fixed nodes, all displacements for the **N2** and **N4** nodes were totally constrained. Degrees of freedom in the **N5** and **N6** nodes were constrained in relation to the direction of axial loads of rod elements (i.e. along

transverse directions). The displacement of nodes **N5** and **N6** depended on the displacement of nodes located in the assumed geometrical dividing planes of the assemblies by implementing a system of rigid links [33, 34]. The lower front rod element constrained by nodes **N9** and **N10** and the lower bottom rod element described by nodes **N7** and **N8** were subjected to tensile forces acting along the axis of symmetry of the spring. To transfer this load to the housing of the inner sleeve of the elastic connector or the inner sleeve in the range exceeding the operating range of the spring, rigid links were implemented [33, 34]. They were connected in the place of division of assemblies with nodes **N8** and **N9**.

Calculating passive and internal forces in the ankle joint assembly nodes, the values of the resultant reactions in the axial and transverse directions for the fixed nodes of the EGI-3 group were determined (Fig. S.5.1.). It was assumed that resultant forces in the longitudinal direction to the axis of symmetry of the elastic connectors equaled predetermined load values of the front connector F_{Fz} and rear connector F_{Bz} (Fig. S.6.3). Similarly, the values of lateral loads acting in the assumed geometrical dividing plane of the front connector F_{Fx} and F_{Fy} and the rear connector F_{Bx} and F_{By} were determined. The concentrated force F_{Fz} was applied at the node **N9**, and the concentrated force F_{Bz} – at the node **N7**. The load from the forces F_{Fx} and F_{Fy} was distributed on nodes located on the surface S_F. The forces F_{Bx} and F_{By} were distributed on nodes associated with the surface S_B. This way of implementation allowed considering an influence of uniaxial forces (tension-compression) and unsymmetric bending.

It is worth paying attention that other loads caused by forces generated by the driving system (Fig. S.3.1) were implemented in the same way as in an ankle joint assembly (Fig. S.5.3). The vectors of concentrated moments T_{1x}, T_{1y}, and T_{1z} were applied at the central node **CN1** and the vector moments T_{3x}, T_{3y}, and T_{3z}, to the node **CN3 (Fig. S.6.3)**.

Nodes discretized mortise area, which was located on a shaft outer surface (15) and underneath the toothed wheel (Fig. 4b), were connected to node **CN1**. This connection modeled an influence of shear (tangential) stress.

S7. FEM model of the hip joint assembly

Considering the mesh model of hip joint assembly (Fig. 8), 9 groups of finite elements were defined (Fig. S.7.1, Table S3). These groups represent the geometric structure of EGIII-1, the connector of the pelvis module, EGIII-2 regulation clamp h_3, EGIII-3 regulation clamp h_5, EGIII-4 connector of the hip joint assembly, EGIII-6 screw of regulation clamp h_3 with its conical head EGIII-5, EGIII-8 screw of regulation clamp h_5 with its conical head EGIII-7, and EGIII-9 mushroom head bolt.

The boundary conditions of surface contact, including penetration and friction [33, 34, 37], were defined by the coefficient of friction μ= 0.1 for pairs shared common surfaces EGIII-1 and EGIII-2, EGIII-2 and EGIII-3, EGIII-3 and EGIII-4, EGIII-2 and EGIII-5, EGIII-4 and EGIII-7. Most of the finite-element groups of the model were described by eight-node finite elements of the 3D-solid type [33, 34].

From a structural point of view, the conical clamping connection between the groups EGIII-2 and EGIII-3 should be modeled by considering a contact zone. The clamp between the elements was

realized by a centrally located screw in the axis of symmetry of the cones. The connection was modeled with a two-node beam element [33, 34]. The extreme nodes of the clamped surfaces located between the head of the screw and the regulation clamp h_3 and between the nut and regulation clamp h_5 (Fig. 5a) were connected in the model to the nodes of the **NB** beams using rigid links (Fig. **S.7.2**) [23, 27]. This allowed introducing an initial loading to the model of the screw (to reflect residual loading). Each screw was modeled by implementing a beam-type element and adapting it in a similar way applied in an ankle joint assembly and knee joint assembly.

The boundary conditions (Fig. **S.7.2**) reflected total fixation between external surface nodes of pelvis segment (EGIII-4) sharing with the surface of part 5 (Fig. 5b).

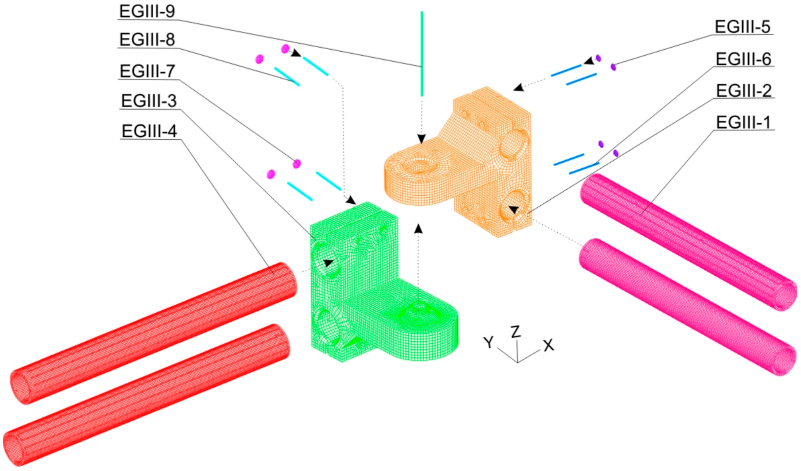

Figure S.7.1. Groups of finite elements of the hip joint assembly.

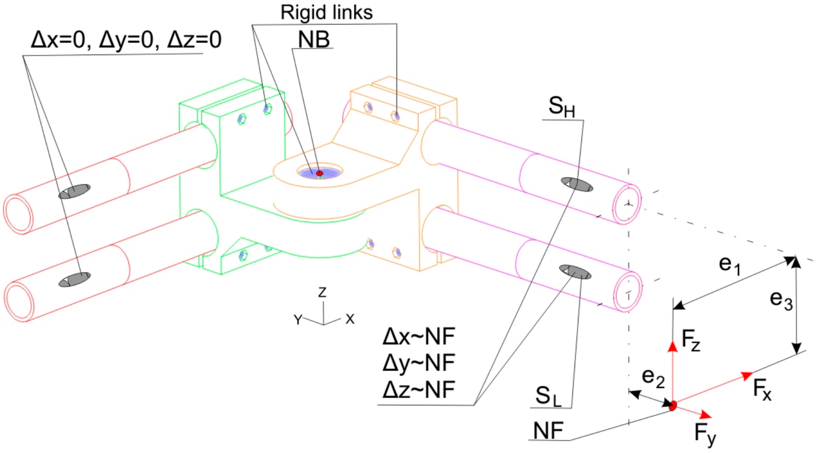

Figure S.7.2. Boundary conditions of the hip joint model.

Aiming to maintain a continuity of internal forces between knee joint assembly and hip joint assembly, external forces were assigned to an additional defined **NF** node. This **NF** node had the same coordinate **z** as nodes **N5** and **N6** (Fig. S.7.2). The remaining coordinates resulted from the eccentric positioning of the elastic connectors (Figs. 4b and S.2.1) described by the dimension e_1 and dimension e_3 (Fig. S.7.2) that were set in relative to the connectors of the knee joint assembly. The loads of nodes located on the outer surfaces (S_H and S_L) of knee joint connectors were coupled to the **NF** node load using rigid links [33, 34]. Assessing passive and internal

forces of nodes of knee joint assembly, the values of the resultant reactions in the axial and transverse directions for EGII-16 group nodes were determined (Figs. S.6.1 and S.6.3). The resultant reaction in the longitudinal directions (nodes **N2** and **N4**) with respect to the symmetry axis of the elastic connectors equaled a value of force F_Z (in the node **NF**, Fig. S.7.2). The resultant component of the reaction of nodes N5 and N6 in the direction of the **x**-axis and the **y**-axis influenced the value of forces F_X and F_Y. Applied implementation allowed considering forces causing skew bending with eccentric stretching.

Table S3. Material model parameters of finite-element groups of the hip joint assembly.

Figure	Part number	Group of finite elements (Fig. S.7.1)	Material	Material model
5a	21	EGIII-2	ABS	Orthotropic
5a	17	EGIII-3		E_a= 900 MPa, E_b = E_c= 1024 MPa<br>ν_{ab}= ν_{ac}=0.457, ν_{bc}= 0.520<br>G_{ab} = G_{ac}= 513 MPa,G_{bc}= 482 MPa<br>R_{am}=26 MPa, R_{bm}=20 MPa, R_{cm}=20 MPa
5a	20	EGIII-1	Polypropylene	Isotropic
5a	19	EGIII-4		E = 2000 MPa<br>ν= 0.3<br>R_e = 35 MPa
5a	18	EGIII-5	Steel	Isotropic
5a	18	EGIII-7		E = 200000 MPa<br>ν = 0.33
5a	18	EGIII-6	Steel	Isotropic
5a	18	EGIII-8		E = 200000 MPa<br>ν = 0.33<br>Circular cross section with the diameter of the screw core d_3 = 4 mm<br>F_B = 1000 N
S.2.1	1	EGIII-16	Steel	Isotropic<br>E = 200000 MPa<br>ν = 0.33<br>Circular cross section with the diameter of the screw core d_3 = 8 mm<br>F_B = 1200 N

S8. FEM results of chosen 3D printed elements

Figs. S.8.1-S8.6 presents distributions of normal stresses σ_a, σ_b, σ_c in regulation clamp h_8 (finite-element group EGI-12) (Fig. 4a) and lower housing of the internal sleeve of the elastic connector (finite-element group EGII-19) (Fig. 4b) along orthogonal directions in State 0, State 1 and State 3.

Considering the regulation clamp h_8 results (Fig. S.8.1), one can define that in ***zone A***, normal stresses exceed the given limit in all orthogonal directions. This may indicate that a proposed pin connection (steel hob and 3D printed element) was not a proper design solution in the case of given loadings. Moreover, screw preload force F_B implemented in State 0 may cause this exceedance along all

orthogonal directions (a, b, c). Analyzing normal stress distributions over State 0 and State 1, one can find: 1) some similarity σ_{amin}= -12 MPa and σ_{amax}= -20 MPa (with quasi-static amplitude $\Delta\sigma_a$= 4MPa) in ***zone B***; 2) that normal stresses exceeded a given limit in *zone C* (Fig. S.8.2).

Analyzing FEM fatigue results of lower housing of the internal sleeve of the elastic connector**,** one can see that: 1) in ***zone A*** and ***zone B*** (Figs. S.8.4- S.8.6), this element sustained one-sided fatigue load and a concentration of normal stress σ_a occurred in the connection of pin with a drive shaft; 2) in ***zone C*** a normal stress distribution was in the range σ_{bmin}= 15 MPa and σ_{bmax}= 18 MPa (with quasi-static amplitude $\Delta\sigma_b$= 1.5 MPa) which was very close to the corresponding results of normal stress σ_c.

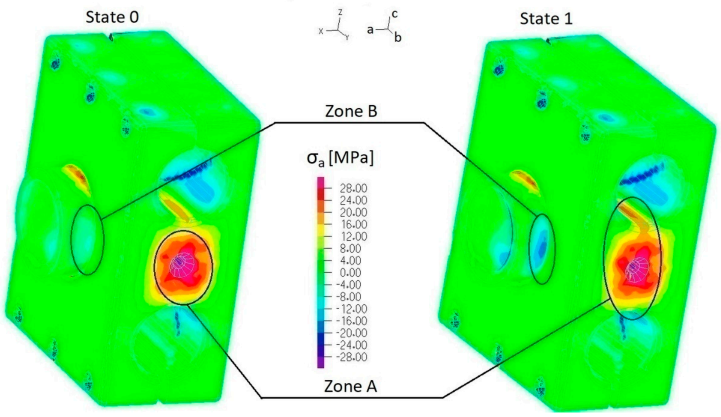

Figure S.8.1. Distribution of normal stresses σ_a in fatigue loaded regulation clamp h_8 (EGI-12) in initial state (State 0) and first state (State 1).

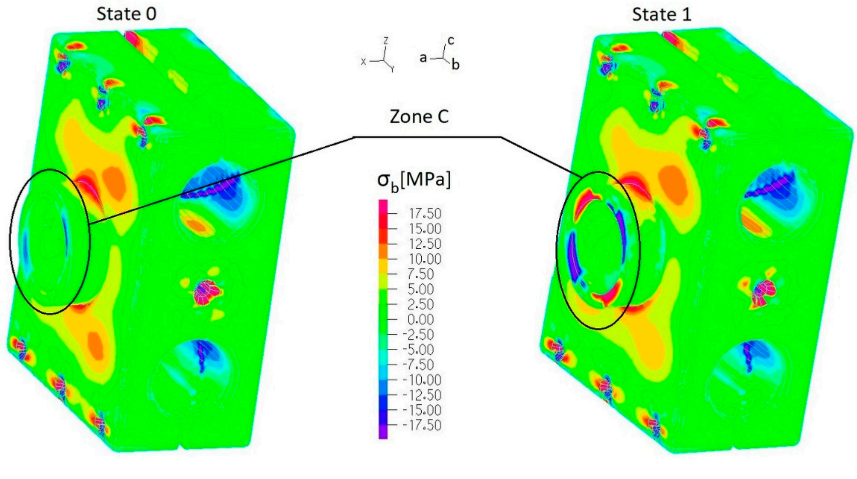

Figure S.8.2. Distribution of normal stresses σ_b in fatigue loaded regulation clamp h_8 (EGI-12) in initial state (State 0) and first state (State 1).

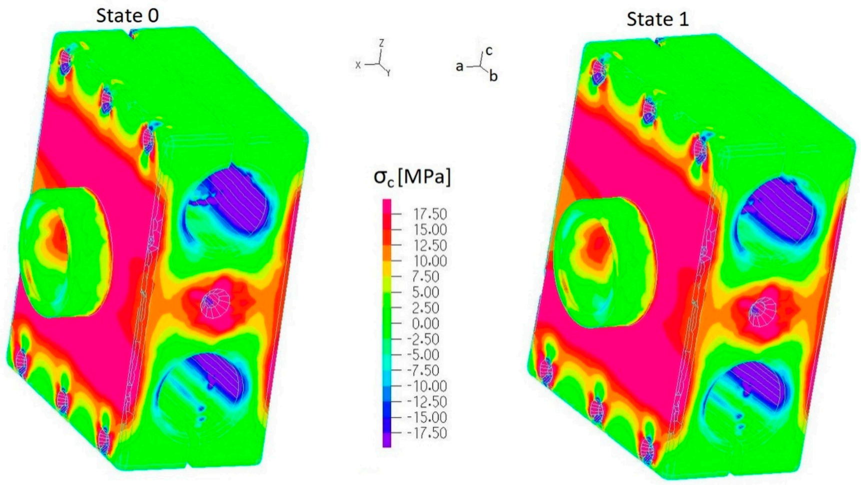

Figure S.8.3. Distribution of normal stresses σ_c in fatigue loaded regulation clamp h_8 (EGI-12) in initial state (State 0) and first state (State 1).

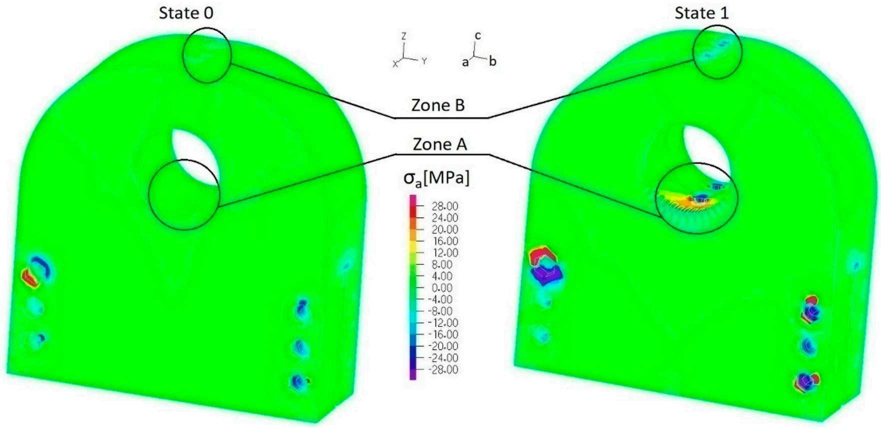

Figure S.8.4. Distribution of normal stresses σ_a in fatigue loaded lower housing of the internal sleeve of the elastic connector (EGII-9) in initial state (State 0) and first state (State 1).

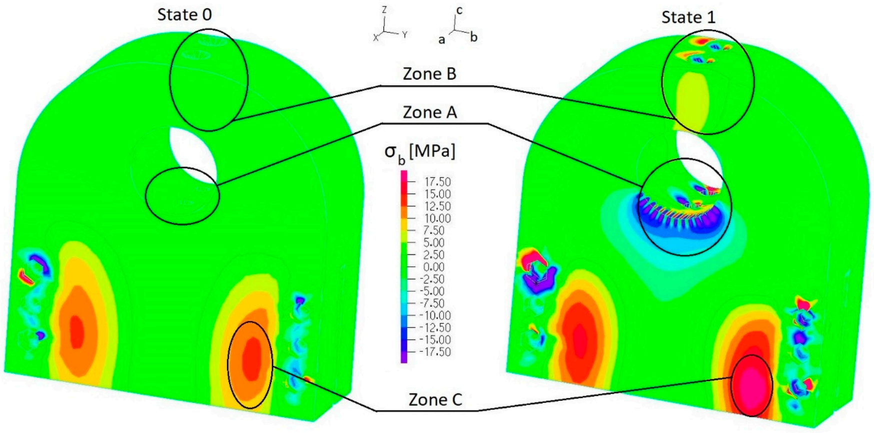

Figure S.8.5. Distribution of normal stresses σ_b in fatigue loaded lower housing of the internal sleeve of the elastic connector (EGII-9) in initial state (State 0) and first state (State 1).

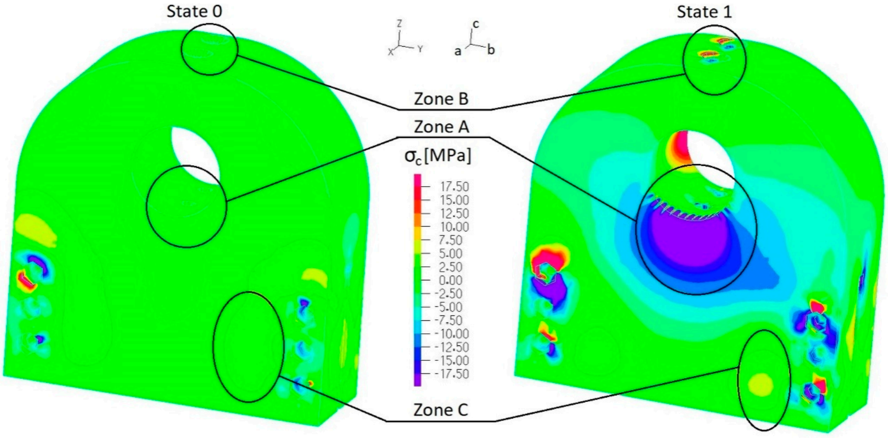

Figure S.8.6. Distribution of normal stresses σ_c in fatigue loaded lower housing of the internal sleeve of the elastic connector (EGII-9) in initial state (State 0) and first state (State 1).
